# Supplementary material for: Variability in interseismic strain accumulation rate and style along the Altyn Tagh Fault
Source: Nat Commun. 2024 Aug 11;15:6876. doi: 10.1038/s41467-024-51116-z (PMC11317509; doi:10.1038/s41467-024-51116-z)
Supplement: Supplementary file 1 — Supplementary Information [file 41467_2024_51116_MOESM1_ESM.pdf]

Supplementary Information for "Variability in interseismic strain accumulation rate and style along the Altyn Tagh Fault"

Lin Shen[1,2*], Andrew Hooper[1], John R. Elliott[1], Tim J. Wright[1]

[1]COMET, School of Earth and Environment, University of Leeds, Woodhouse Lane, Leeds, LS2 9JT, United Kingdom.
[2]Lamont-Doherty Earth Observatory, Columbia University, 61 Rte 9W, Palisades, New York, 10964, United States of America.

*Corresponding author(s). E-mail(s): lshen@ldeo.columbia.edu;

Contents

1. Supplementary Figures 1 to 18

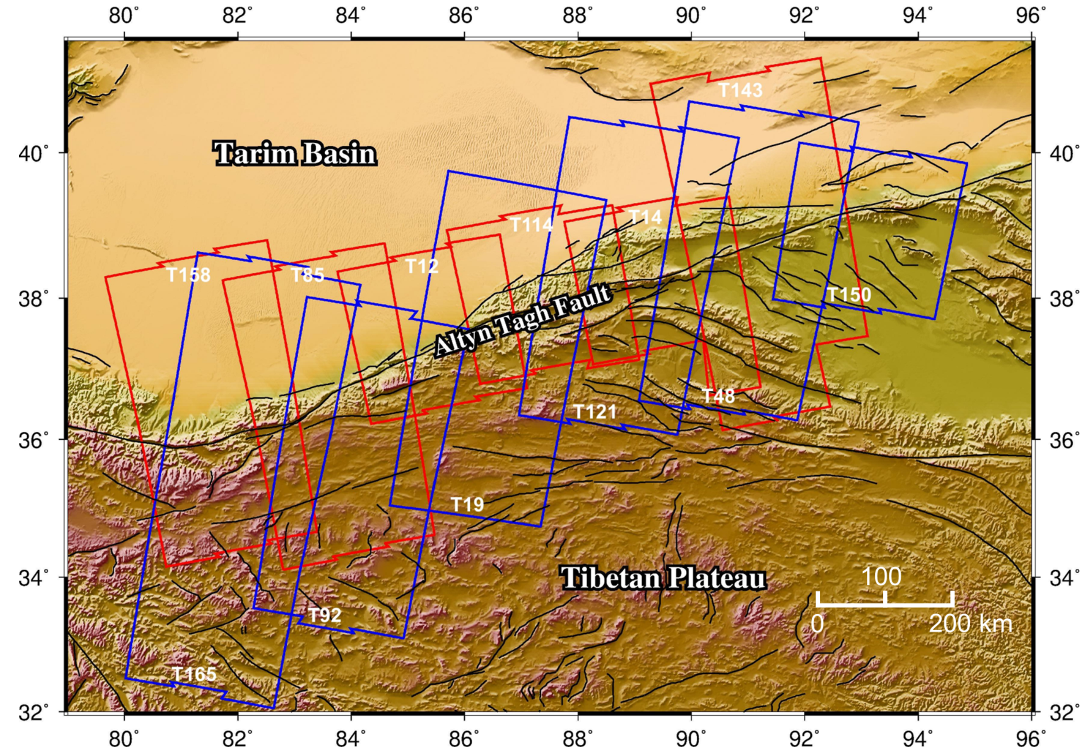

Supplementary Fig. 1: Sentinel-1 data tracks across the Altyn Tagh Fault, including 6 tracks in ascending (red polygons) and 6 tracks in descending (blue polygons). The map background shows the elevation of the study region derived from the Shuttle Radar Topography Mission (SRTM) 3-arc seconds data.

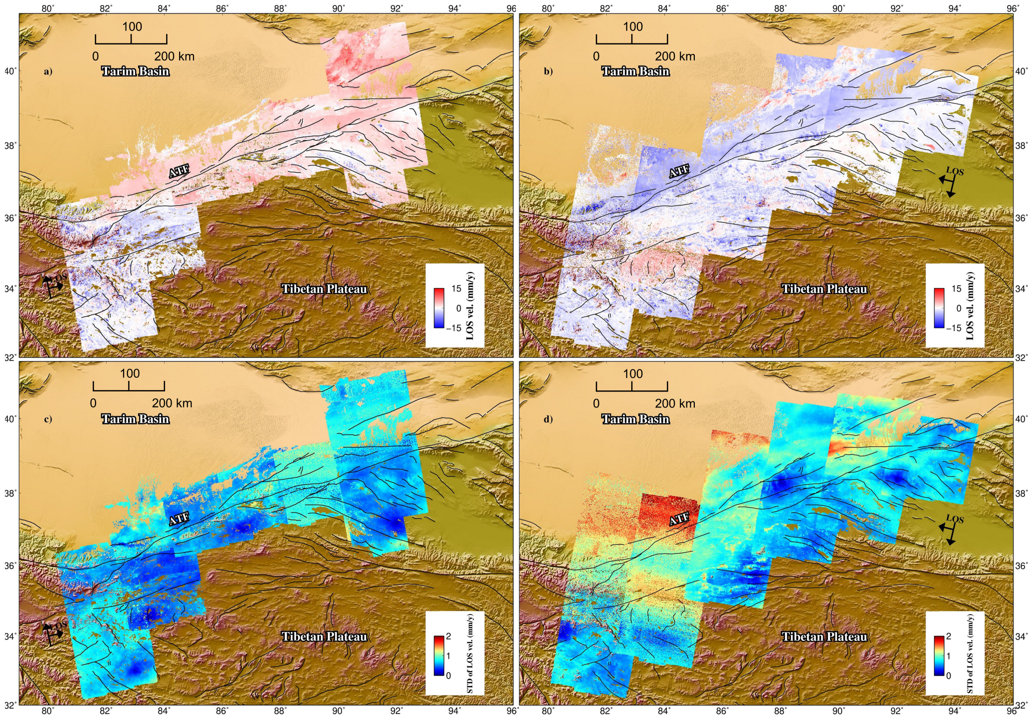

Supplementary Fig. 2: Mosaicked InSAR line-of-sight (LOS) velocity fields in ascending (a) and descending (b) after removing the average offsets in overlapping areas. Positive values indicate the motion towards the satellite, whereas negative values show the motion away from the satellite. (c) and (d) are the estimated standard deviation of velocities accordingly using the percentile bootstrap method. ATF = Altyn Tagh Fault. The map background shows the elevation of the study region derived from the Shuttle Radar Topography Mission (SRTM) 3-arc seconds data.

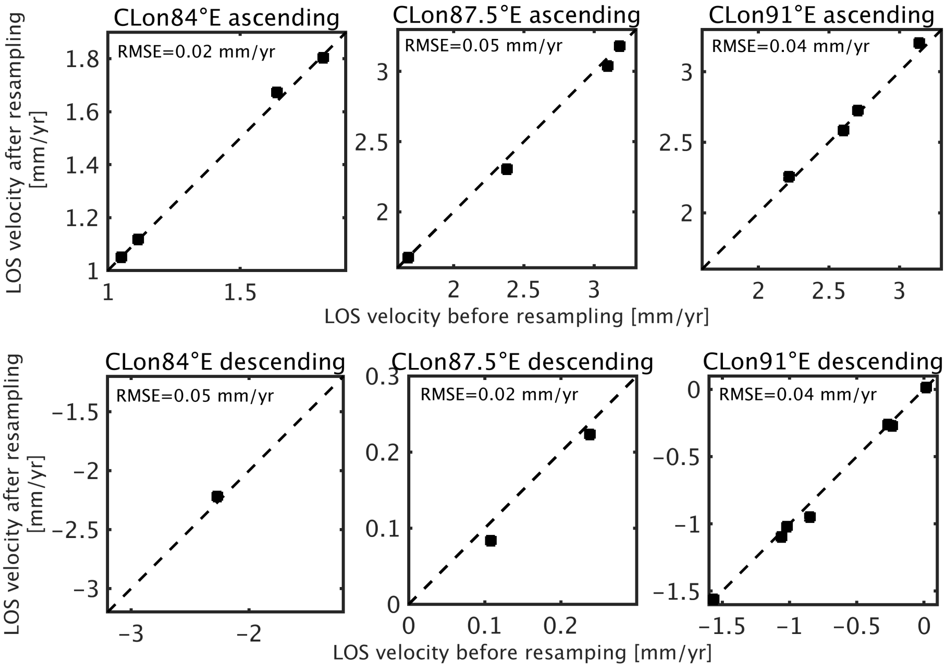

Supplementary Fig. 3: InSAR line-of-sight (LOS) velocities before and after resampling along short fault-parallel velocity profiles with a length of 10 km on either side of the Altyn Tagh Fault at 84°E, 87.5°E, and 91°E, respectively, in the ascending (top panels) and descending (bottom panels) directions. Proximity to the black dashed line, which marks equality, implies that velocities are consistent before and after resampling.

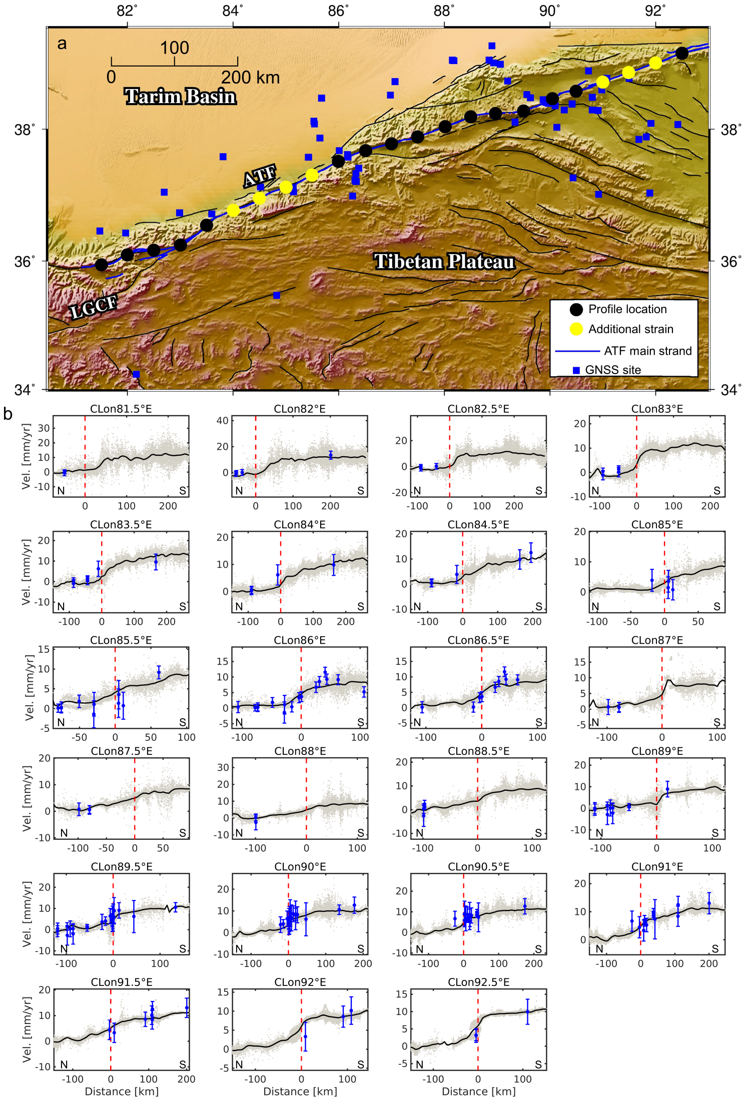

Supplementary Fig. 4: Fault-parallel velocity profiles of the Altyn Tagh Fault (ATF) derived from the line-of-sight (LOS) velocity field. In the upper panel (a), the black and yellow points indicate the fault location of each profile on the high-resolution fault trace of the ATF (blue lines). The yellow points indicate locations for which the profiles show additional strain localisation south of the ATF. The map background shows the elevation of the study region derived from the Shuttle Radar Topography Mission (SRTM) 3-arc seconds data. LGCF = Longmu-Gozha Co Fault. In the profile panels (b), the grey points are the mean fault-parallel velocities of points projected from within a 50 km perpendicular distance onto each profile; the black bold line represents the mean velocities binned by every 5 km along the profile; the red dashed lines show the mapped fault locations of main ATF; the blue points are the resolved fault-parallel GNSS velocities projected onto the profile with $\pm 2\sigma$ errorbars. Vel. = Velocity.

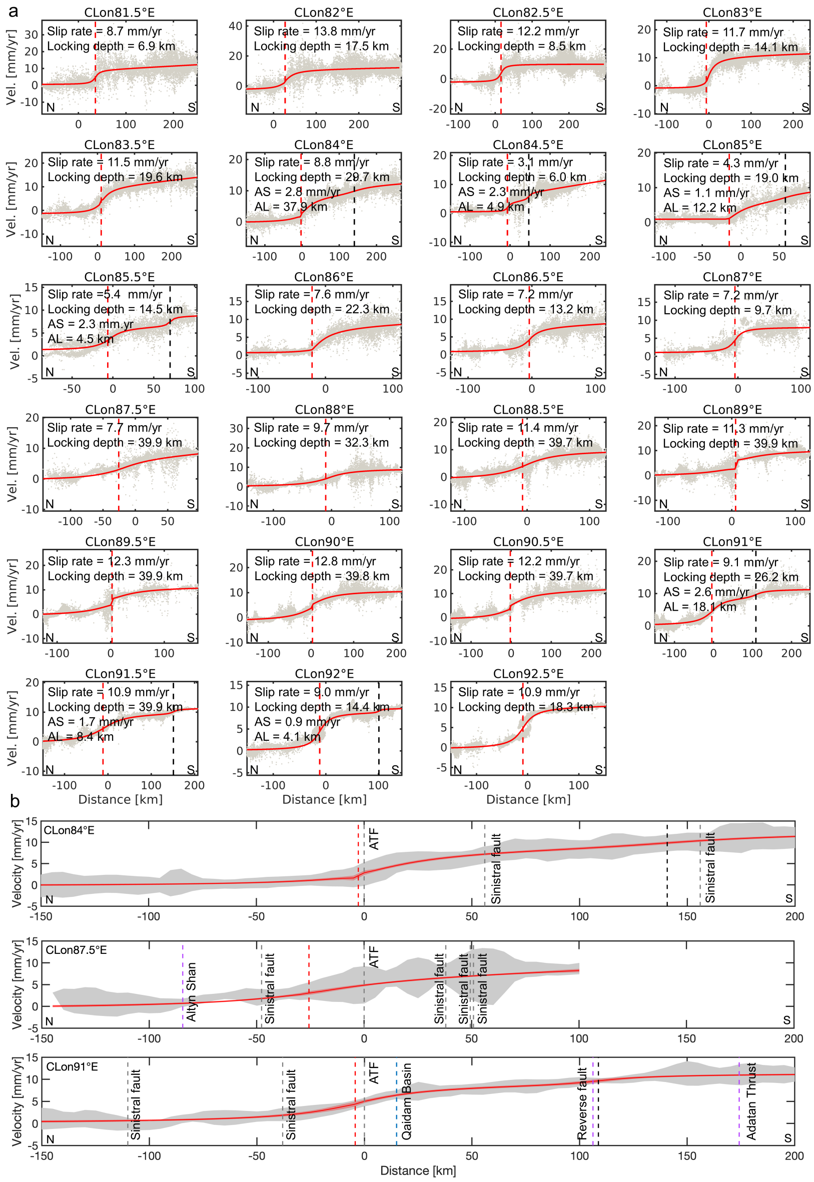

Supplementary Fig. 5: The maximum a posteriori (MAP) solution based on a modified deep-fault model. (a) shows the MAP solution for each fault-perpendicular profile along the Altyn Tagh Fault (ATF) as red curves. Red dashed lines represent the estimated locations of the buried dislocations along the ATF. Black dashed lines show the estimated locations of the buried dislocation of the additional strain on these profiles. (b) shows three enlarged profiles at 84°E, 87.5°E and 91°E, respectively. The light red-shaded region represents the 95% model confidence range. Grey dashed lines are the locations of mapped geological structures intersecting with each profile. Vel. = Velocity. AS = Additional slip rate on secondary faults. AL = Additional locking depth on secondary faults.

Supplementary Fig. 6: Marginal probability distributions of each profile from 81.5°E to 92.5°E (a to w) along the Altyn Tagh Fault (ATF) based on a modified deep-fault model. Abbreviations of labels include 'S rate (Slip rate)', 'L depth (Locking depth)', 'H shifts (Horizontal shifts)', 'S offset (Static offset)', 'R ratio (Rigidity ratio)', 'C L depth (Creeping depth)', 'C rate (Creep rate)', 'R rate (Rotation rate)', 'A L depth (Additional locking depth)','A S rate (Additional slip rate)' and 'A H shifts (Additional horizontal shifts)'.

(a) At 81.5°E.

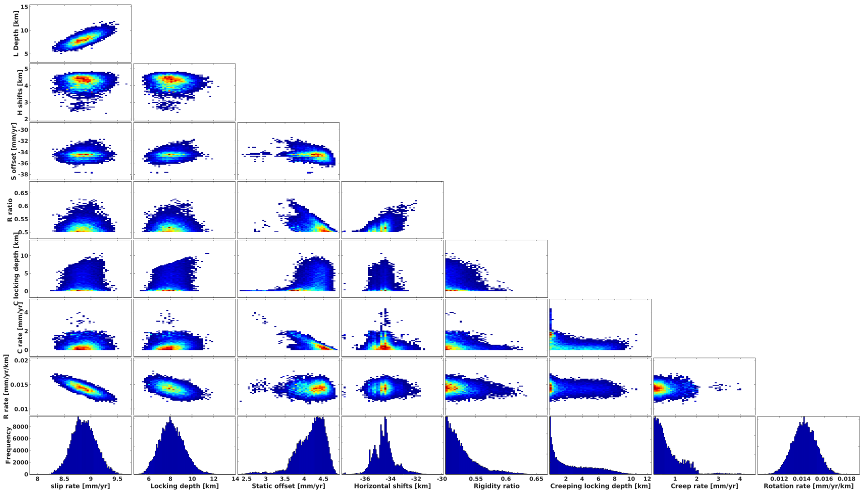

(b) At 82 °E.

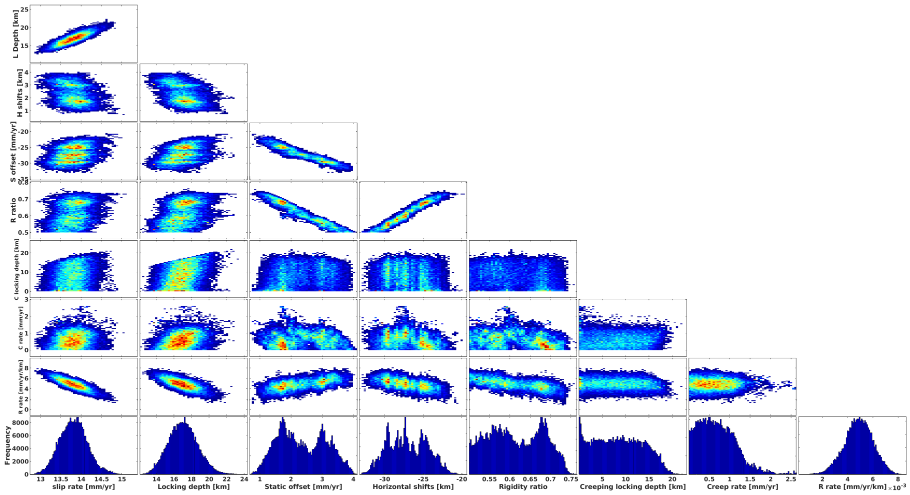

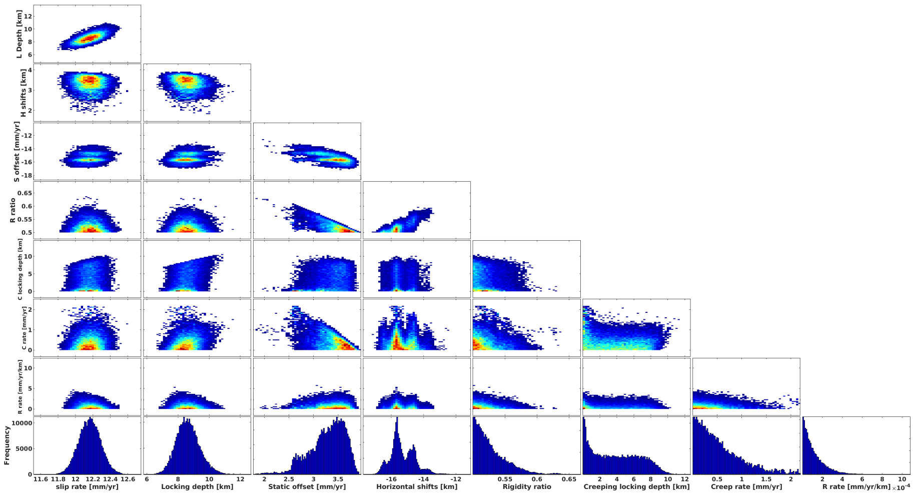

(c) At 82.5°E.

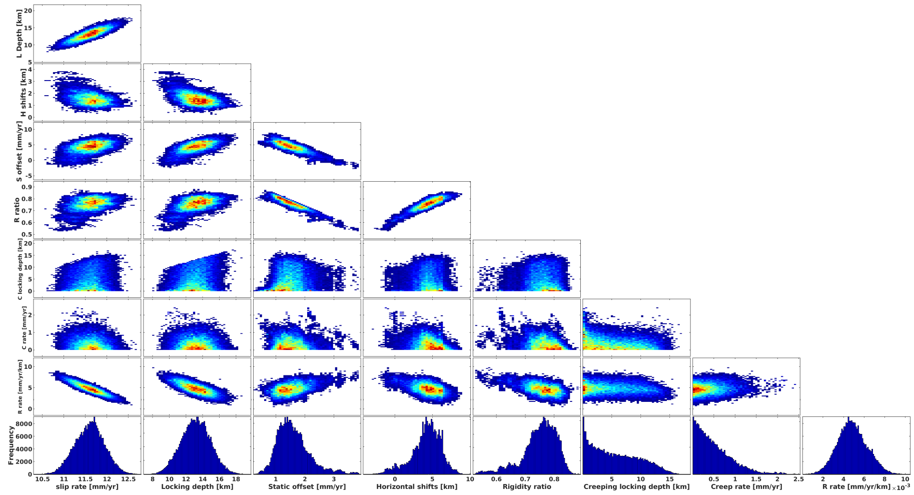

(d) At 83°E.

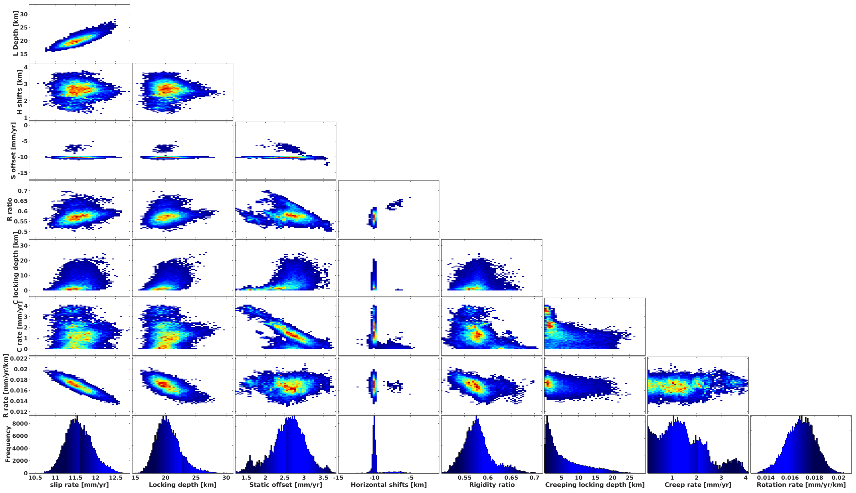

(e) At 83.5°E.

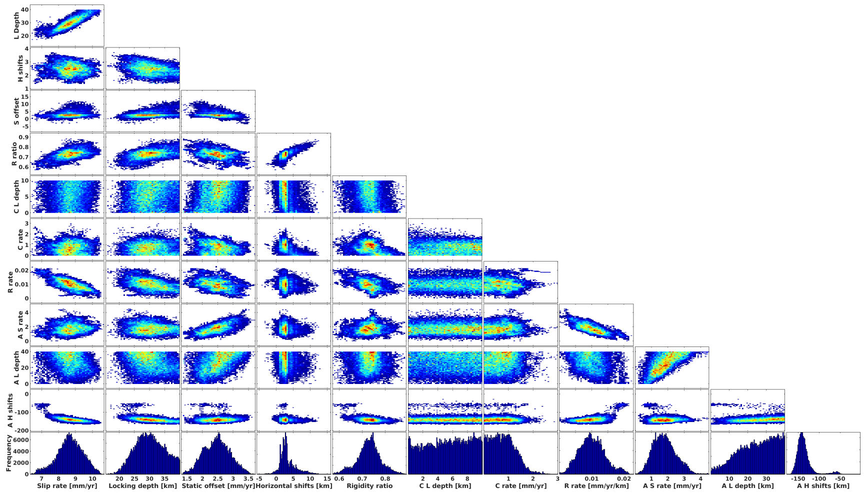

(f) At 84°E.

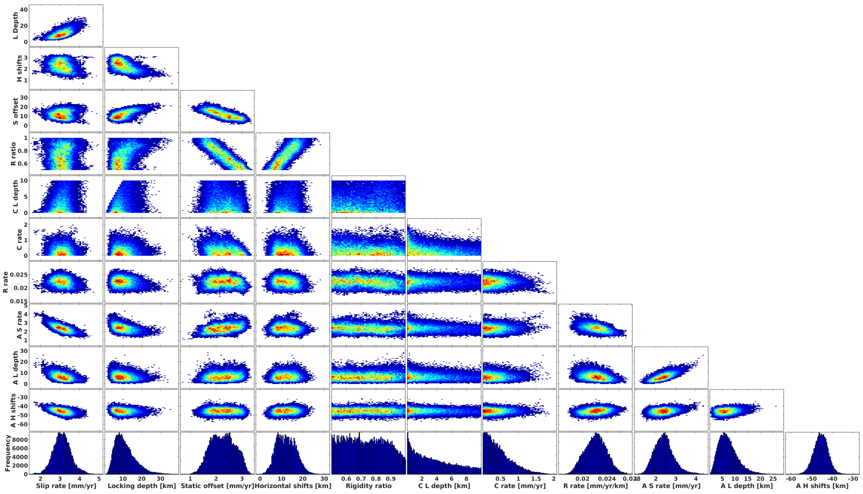

(g) At 84.5°E.

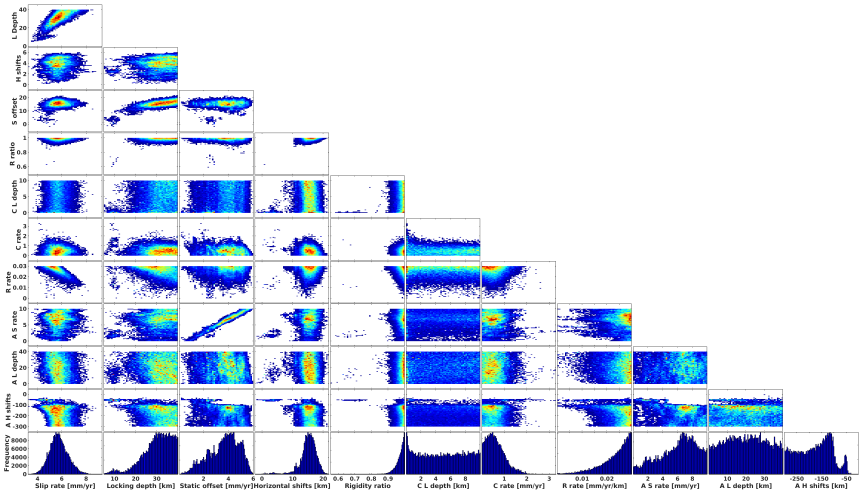

(h) At 85°E.

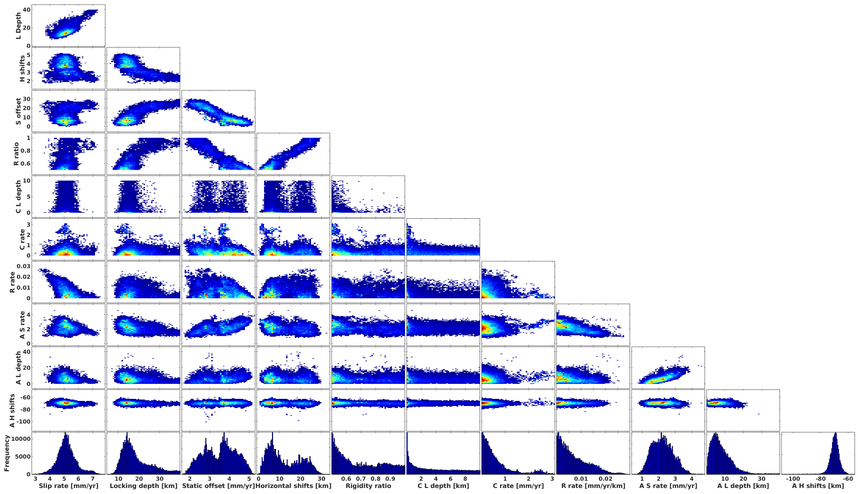

(i) At 85.5°E.

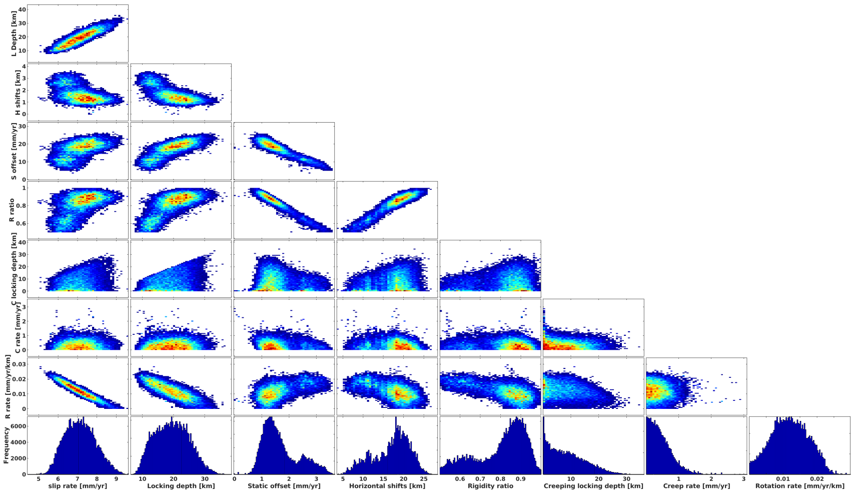

(j) At 86°E.

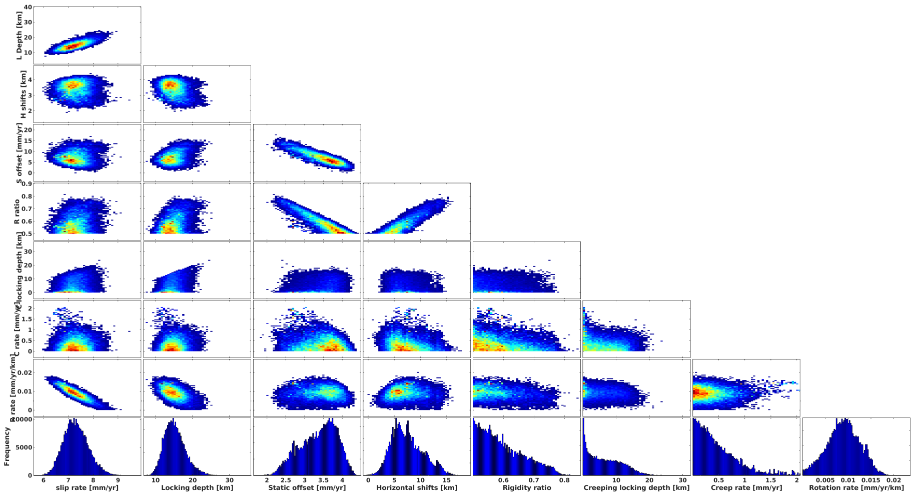

(k) At 86.5°E.

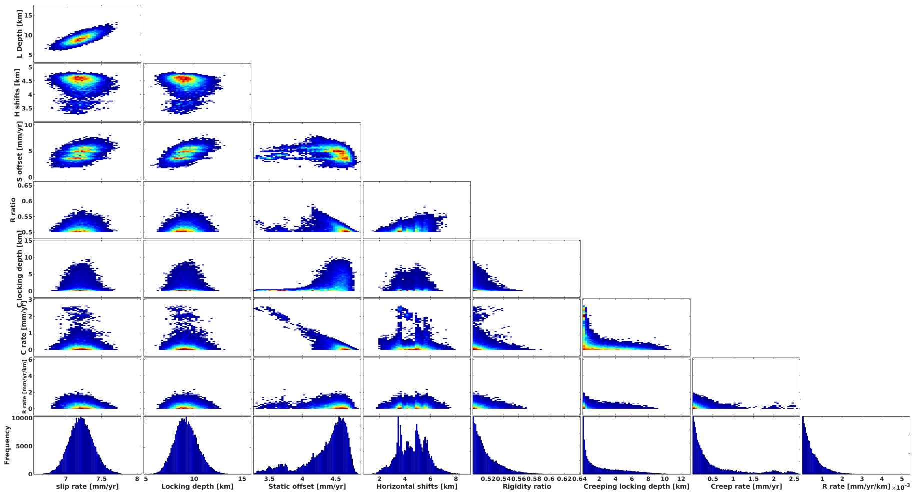

(l) At 87°E.

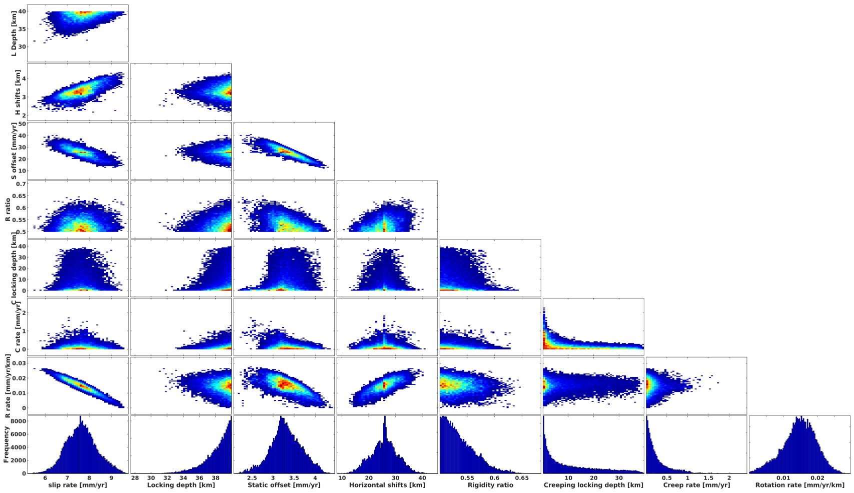

(m) At 87.5°E.

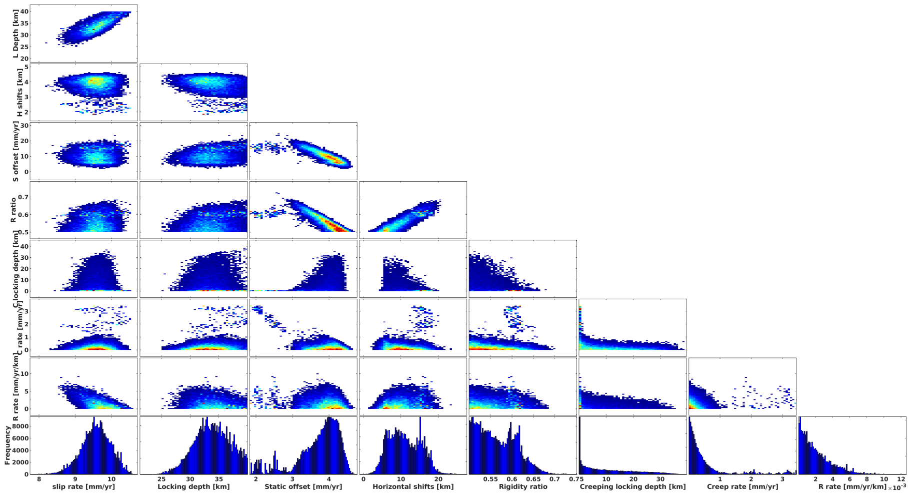

(n) At 88°E.

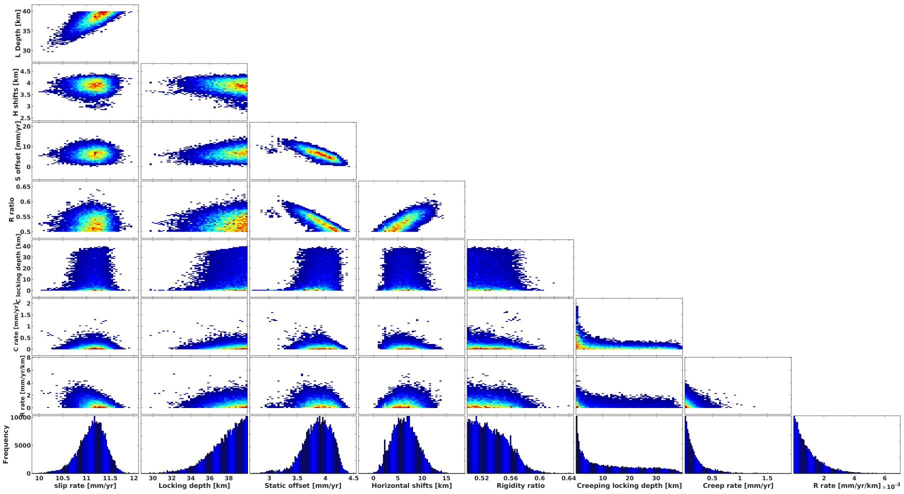

(o) At 88.5°E.

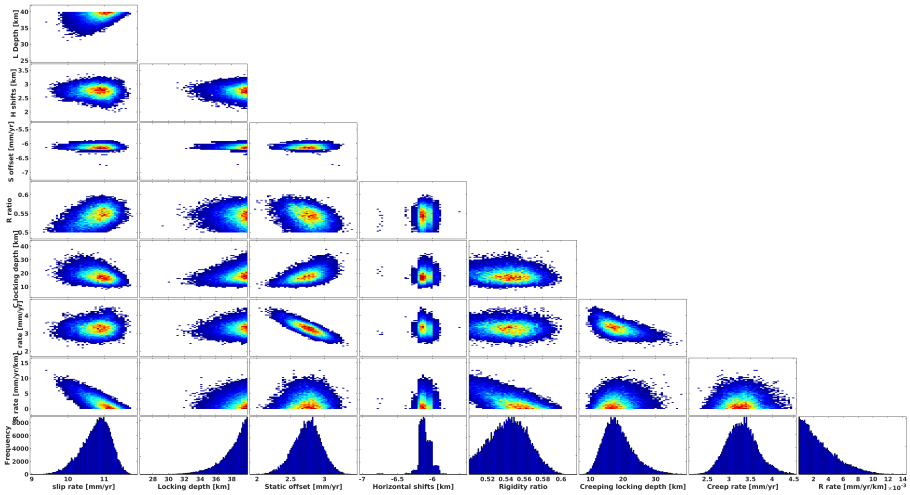

(p) At 89°E.

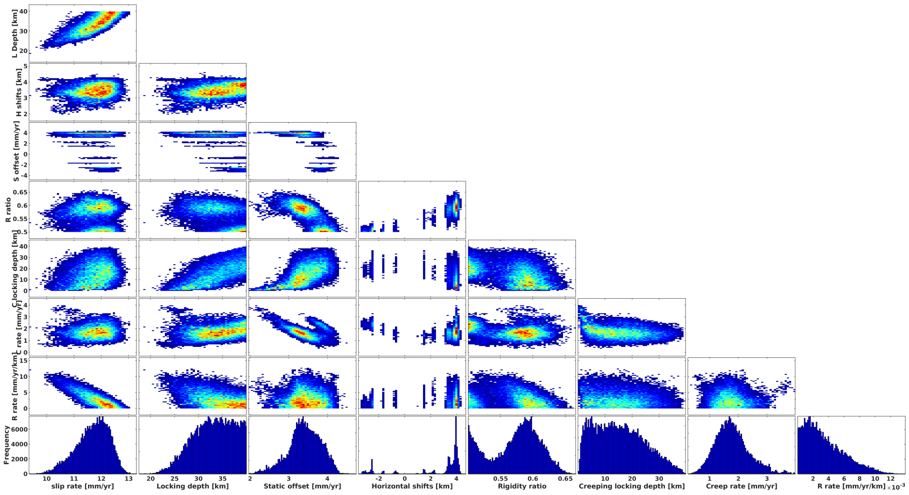

(q) At 89.5°E.

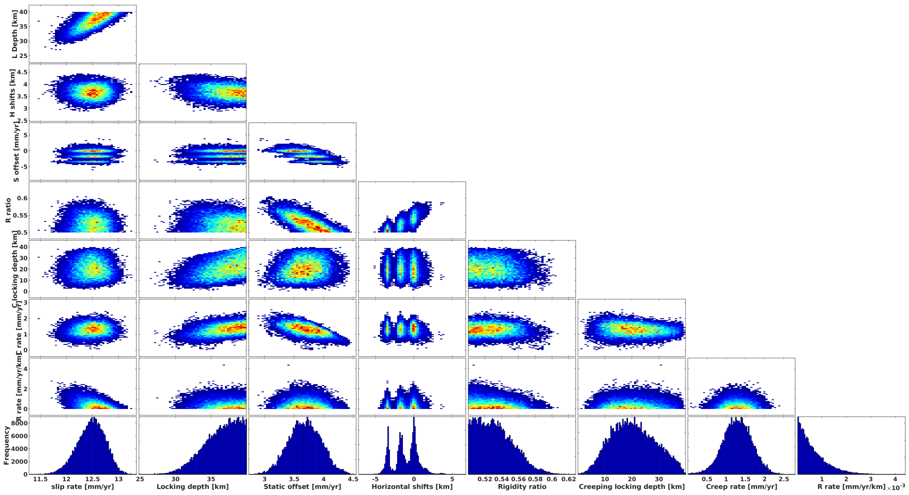

(r) At 90°E.

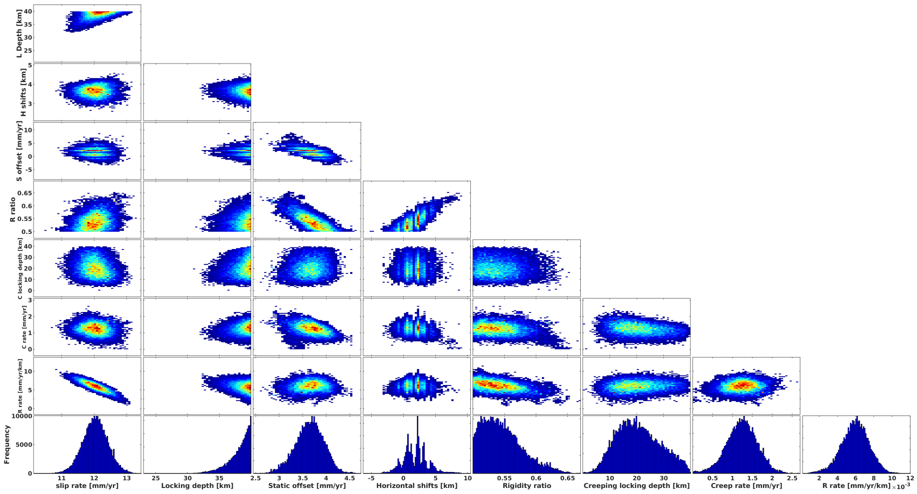

(s) At 90.5°E.

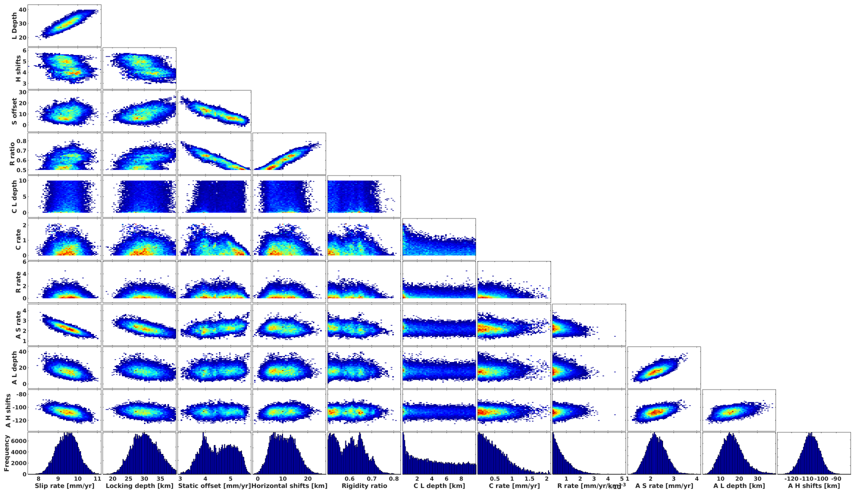

(t) At 91°E.

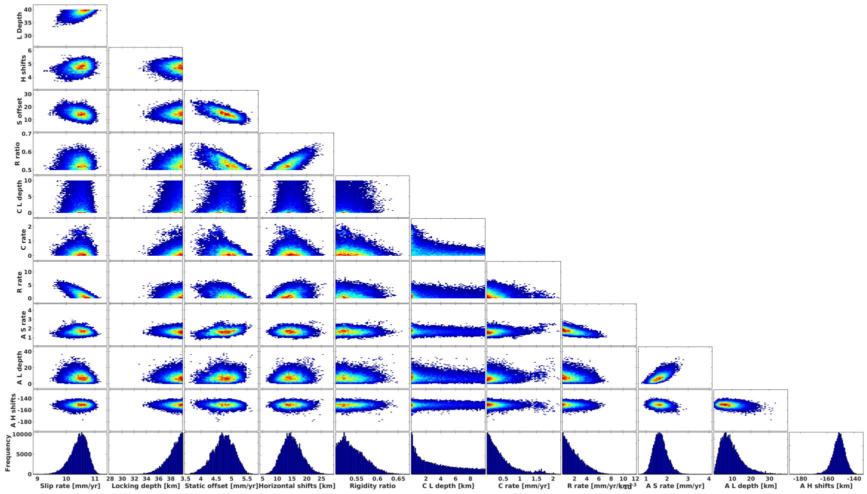

(u) At 91.5°E.

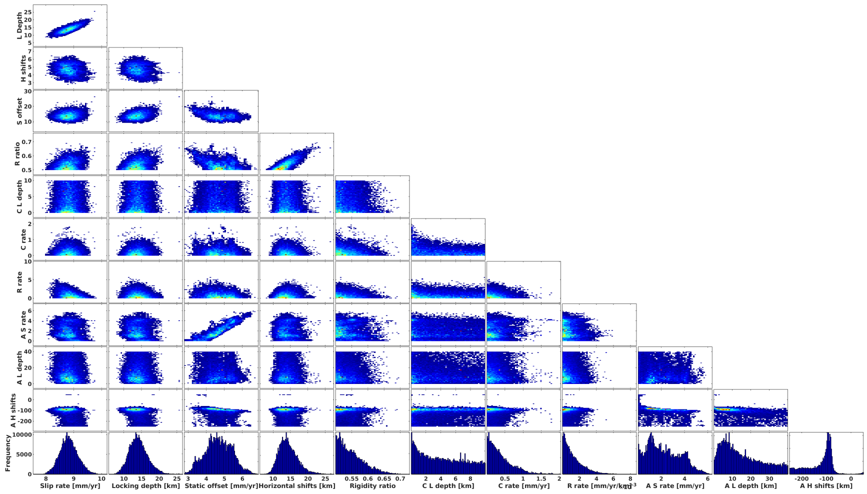

(v) At 92°E.

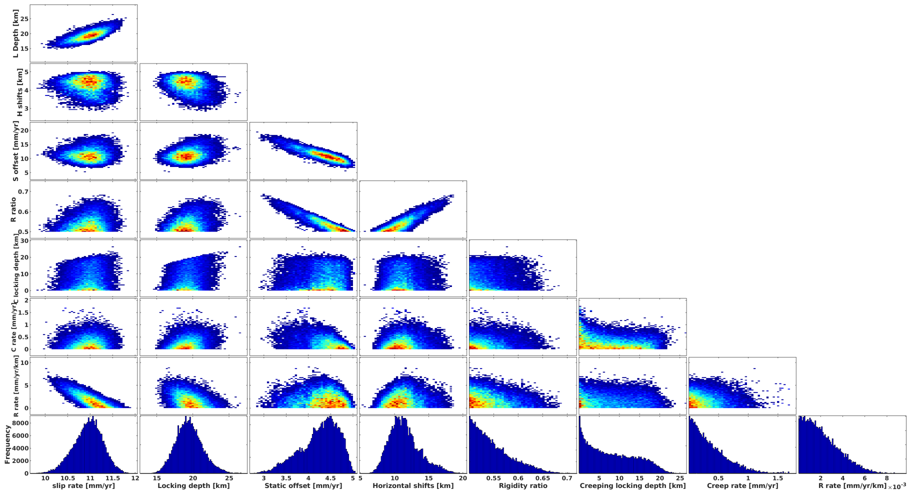

(w) At 92.5°E.

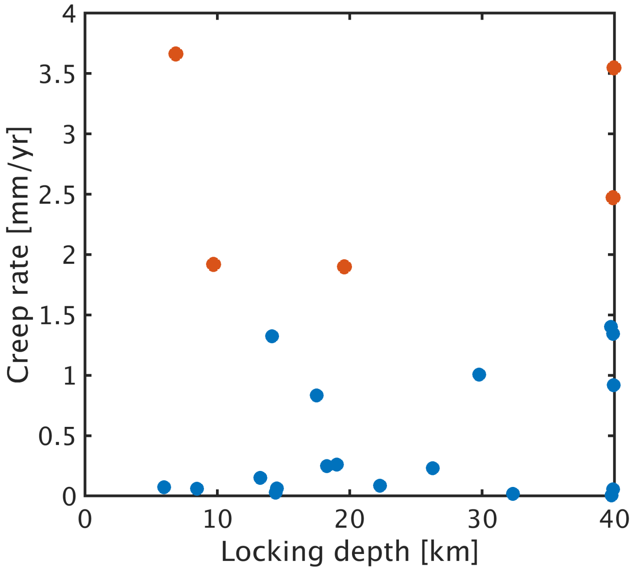

Supplementary Fig. 7: Estimated locking depth against estimated creep rate along the Altyn Tagh Fault. Locations with creep rates higher than 1.5 mm/yr are shown in red.

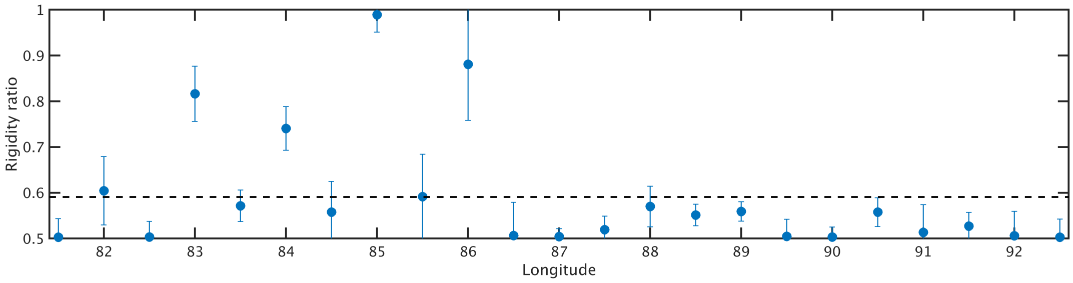

Supplementary Fig. 8: Estimates of rigidity ratio between the Tarim Basin and the Tibetan Plateau along the Altyn Tagh Fault. The error bars represent the 68% confidence bounds on the estimates. The black dashed line gives the average estimate.

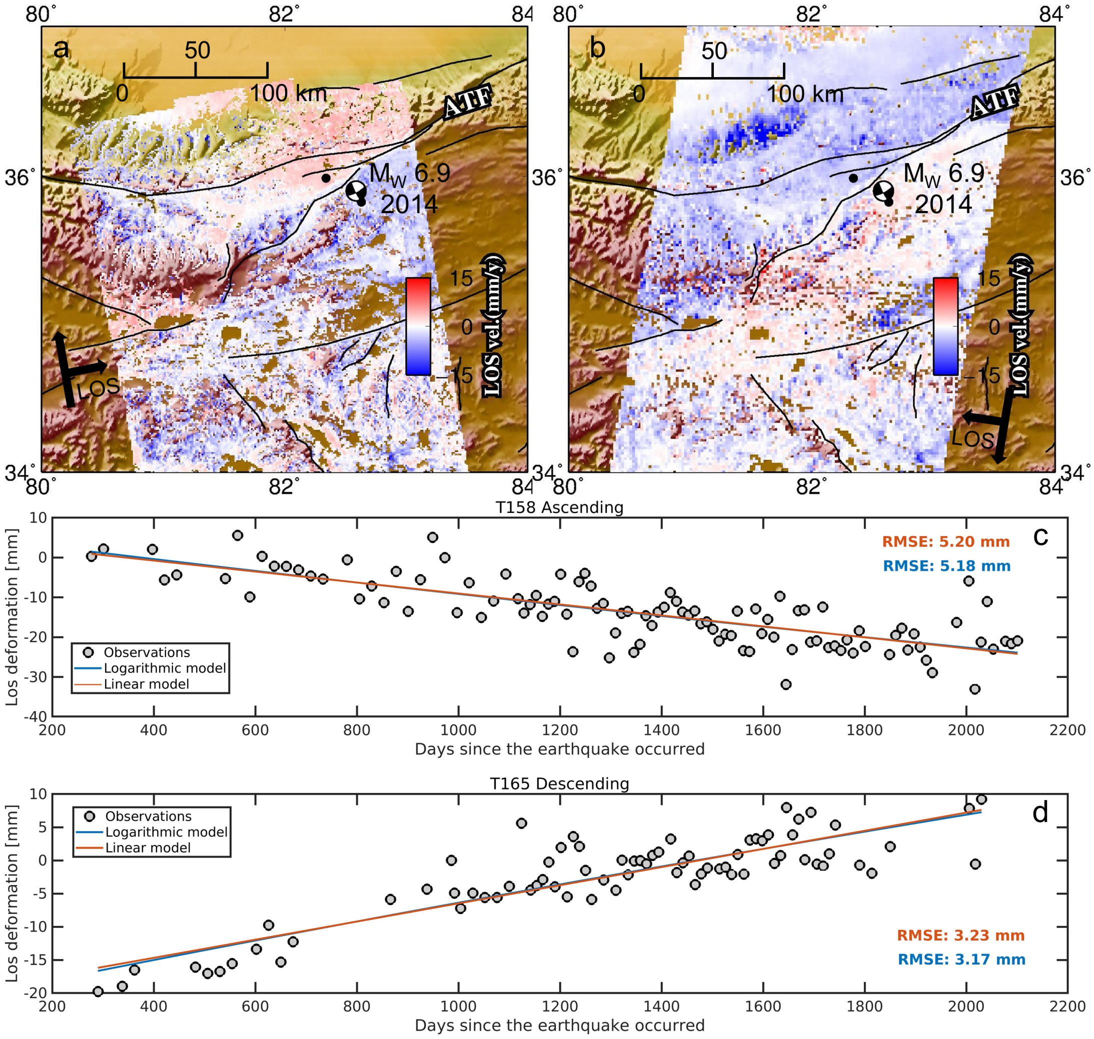

Supplementary Fig. 9: Temporal evolution of line-of-sight (LOS) displacement between two sites on either side of the south-western segment of the Altyn Tagh Fault (ATF). (a) and (b) show the InSAR LOS velocity fields of the ascending track 158 and the descending track 165, respectively. Black points in (a) and (b) represent the locations of two sites. The focal mechanism indicates the February 2014 M_w 6.9 Yutian earthquake. The map background shows the elevation of the study region derived from the Shuttle Radar Topography Mission (SRTM) 3-arc seconds data. Black-gray scatters in (c) and (d) show the time series of the relative LOS displacement between the two sites for the ascending and descending track, respectively. Blue and orange lines represent the best fit logarithmic and linear model to the time series, respectively.

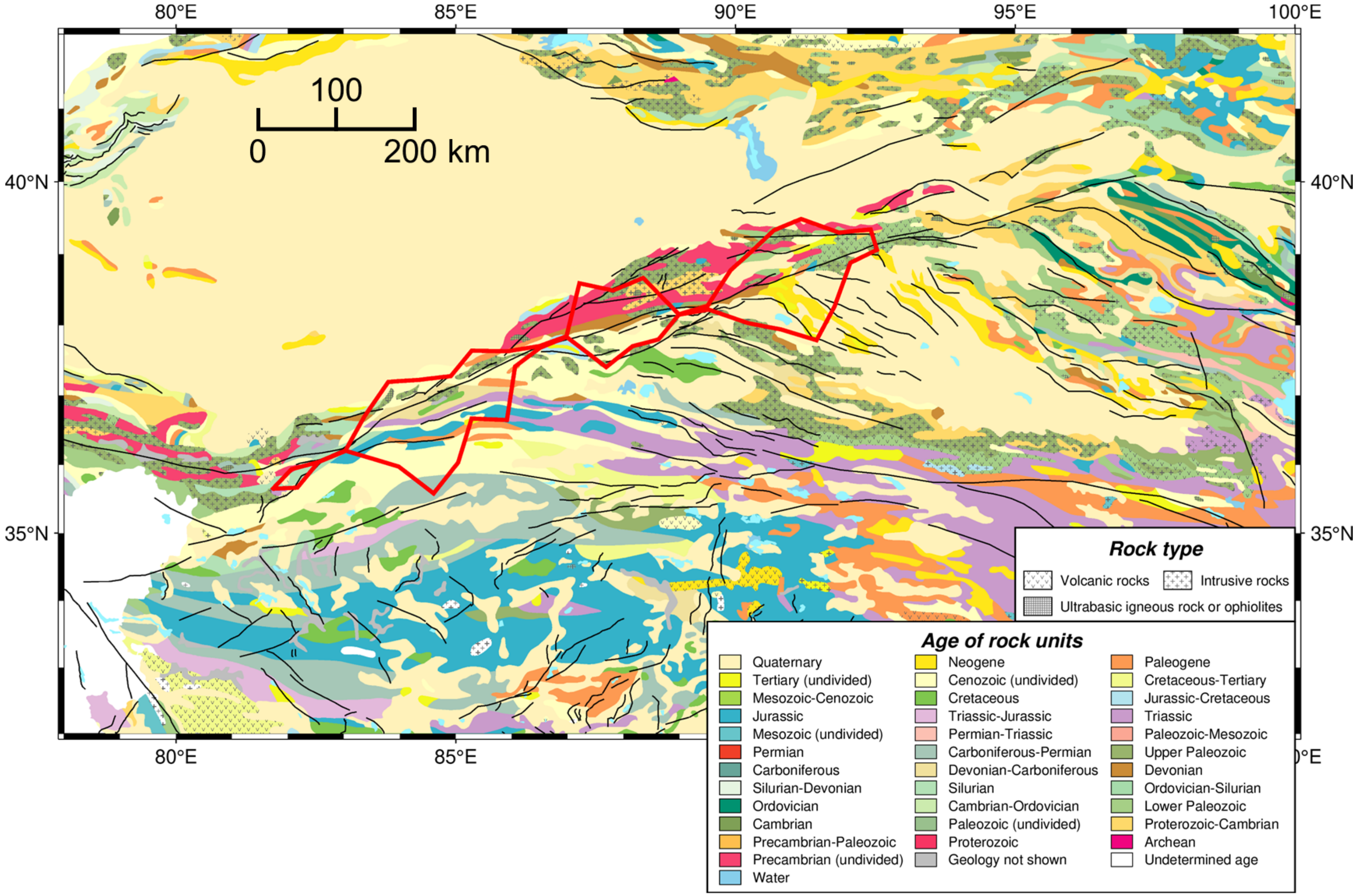

Supplementary Fig. 10: Comparison of the estimated shear zones along the Altyn Tagh Fault (ATF) with the distribution of geologic age and bedrock surface outcrops in the Northern Tibetan Plateau. Solid red lines represent the locations of the estimated shear zones along the ATF. The geological map background is publicly available from the ScienceBase-Catalog of the United States Geological Survey (https://www.sciencebase.gov/catalog/item/60abc7f9d34ea221ce51e5ee).

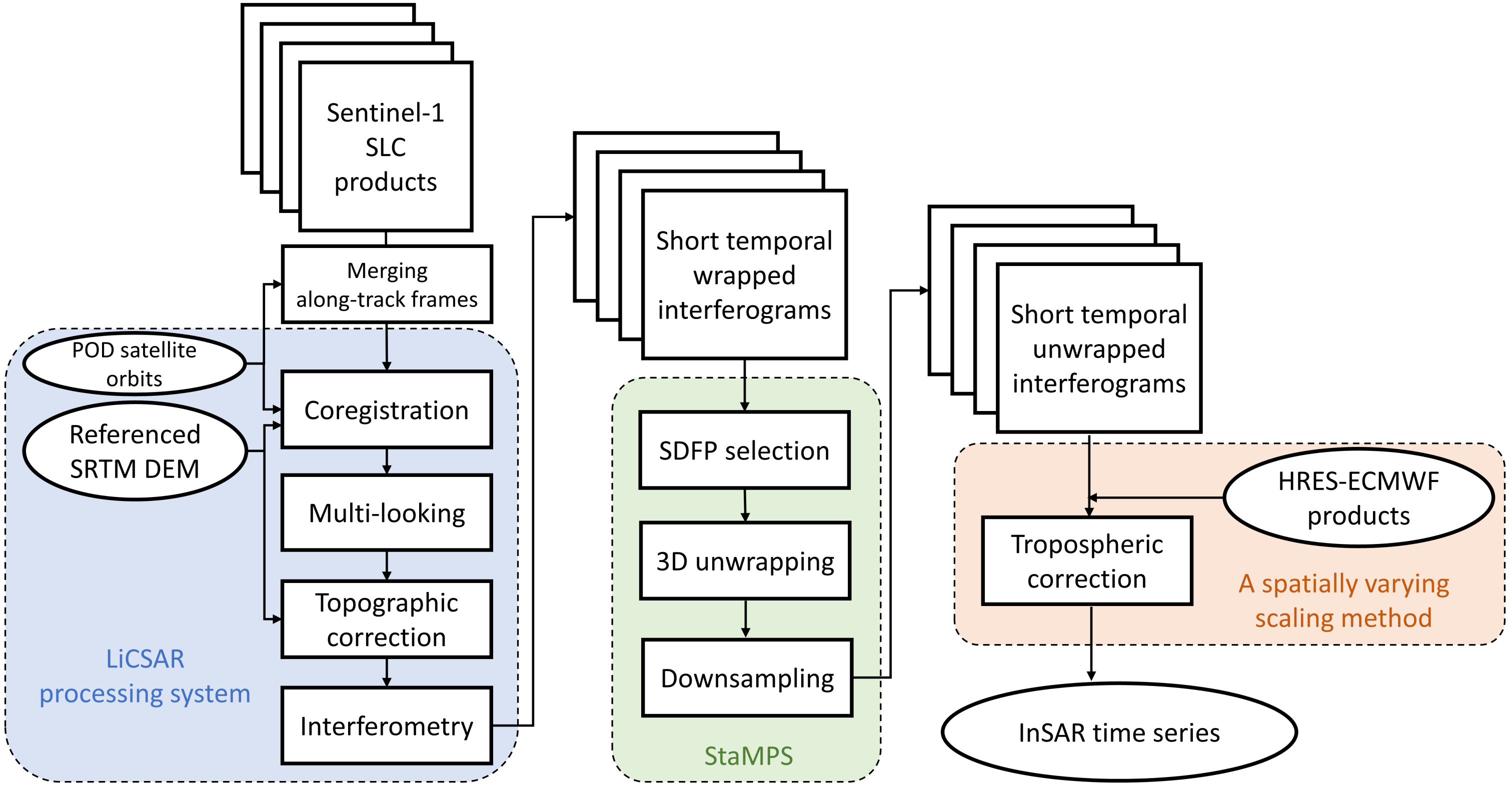

Supplementary Fig. 11: A work flow diagram illustrating the main steps of InSAR processing from Sentinel-1 Simple Look Complex (SLC) products to time-series.

Supplementary Fig. 12: Small baseline subset networks of the individual tracks, including 6 descending tracks (a to f) and 6 ascending tracks (g to l). The red circles represent the epochs of SAR acquisitions and the black lines indicate the generated small temporal baseline interferograms. Bperp = Perpendicular baseline.

(a)

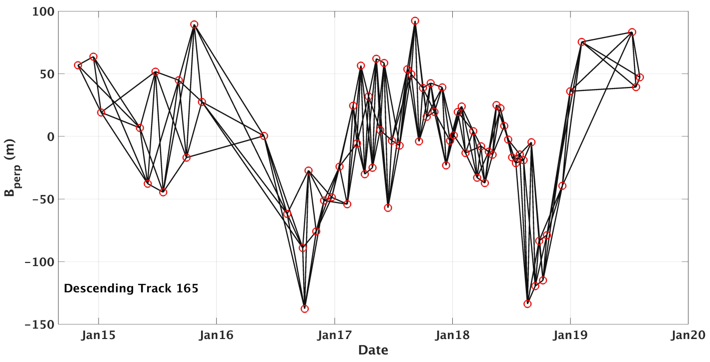

(b)

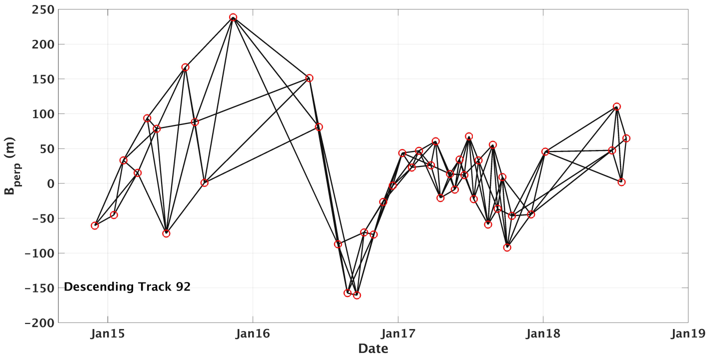

(c)

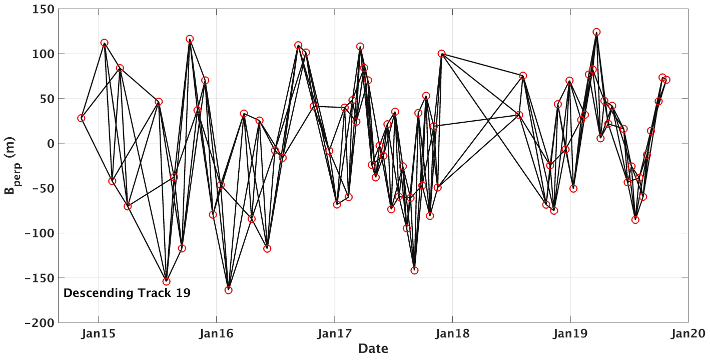

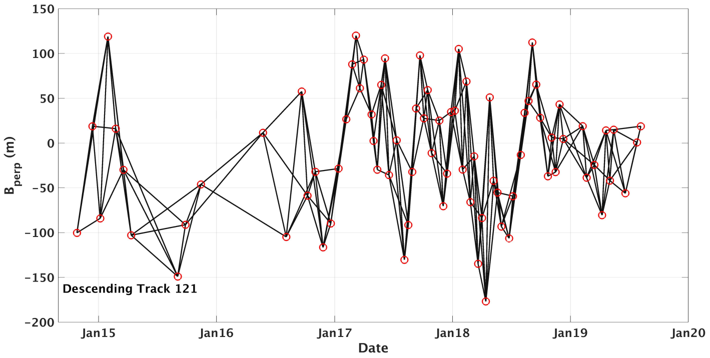
150
100
50
0
-50
-100
-150
-200
B_{perp} (m)
Descending Track 121
Jan15
Jan16
Jan17
Jan18
Jan19
Jan20
Date

(d)

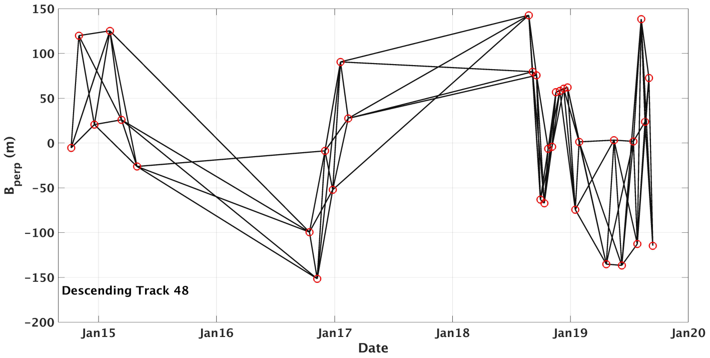
150
100
50
0
-50
-100
-150
-200
B_{perp} (m)
Descending Track 48
Jan15
Jan16
Jan17
Jan18
Jan19
Jan20
Date

(e)

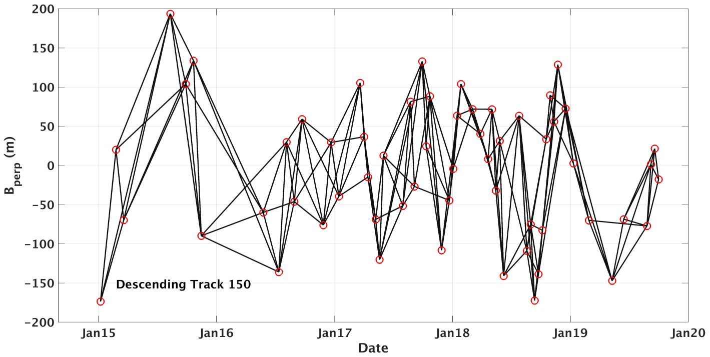
200
150
100
50
0
-50
-100
-150
-200
B_{perp} (m)
Descending Track 150
Jan15
Jan16
Jan17
Jan18
Jan19
Jan20
Date

(f)

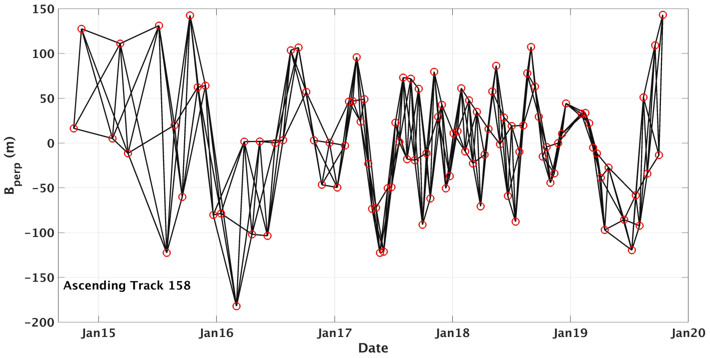

Ascending Track 158
B_perp (m)
Date
Jan15
Jan16
Jan17
Jan18
Jan19
Jan20
150
100
50
0
−50
−100
−150
−200

(g)

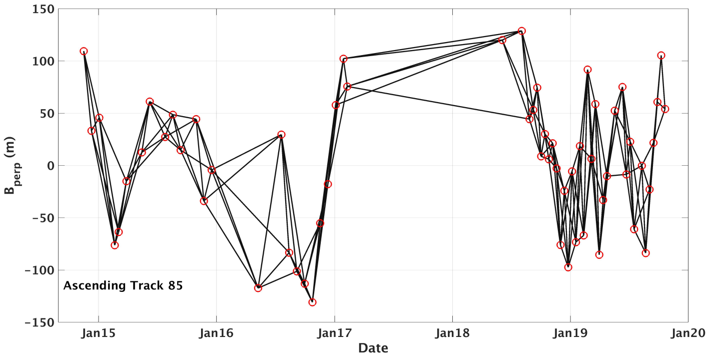

Ascending Track 85
B_perp (m)
Date
Jan15
Jan16
Jan17
Jan18
Jan19
Jan20
150
100
50
0
−50
−100
−150

(h)

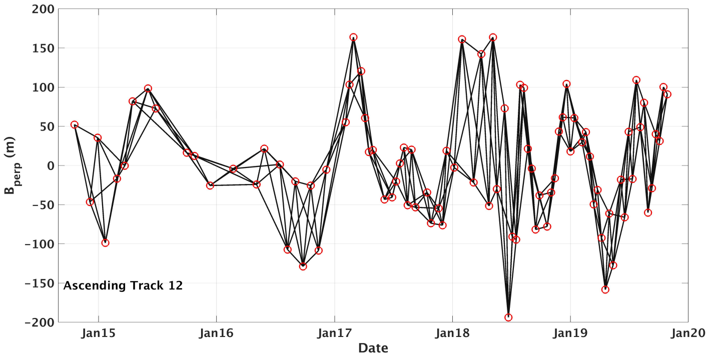

Ascending Track 12
B_perp (m)
Date
Jan15
Jan16
Jan17
Jan18
Jan19
Jan20
200
150
100
50
0
−50
−100
−150
−200

(i)

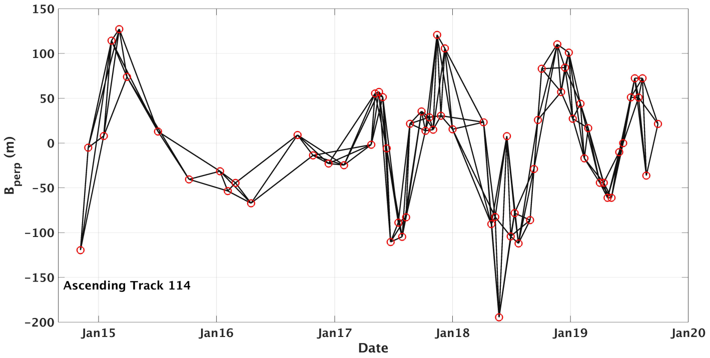
Ascending Track 114
B perp (m)
Date
Jan15
Jan16
Jan17
Jan18
Jan19
Jan20

(j)

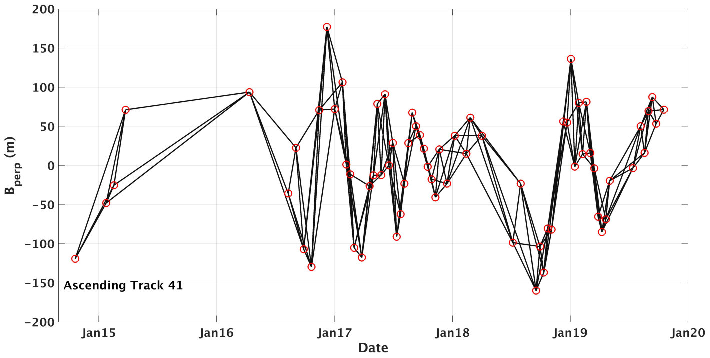
Ascending Track 41
B perp (m)
Date
Jan15
Jan16
Jan17
Jan18
Jan19
Jan20

(k)

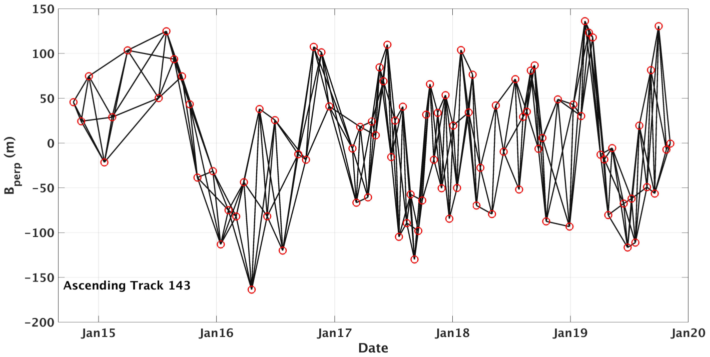
Ascending Track 143
B perp (m)
Date
Jan15
Jan16
Jan17
Jan18
Jan19
Jan20

(l)

Supplementary Fig. 13: Comparisons of interferograms for individual tracks before and after tropospheric corrections: Histograms of the InSAR phase delays versus topography for epochs that are strongly influenced by tropospheric delays, before and after tropospheric corrections (a to l). The black lines are the best fitting linear function, shown for reference. All tracks show a reduction in the root-mean-square (RMS) after the correction is applied (m).

(a) Track 165: 6 Sept 2015

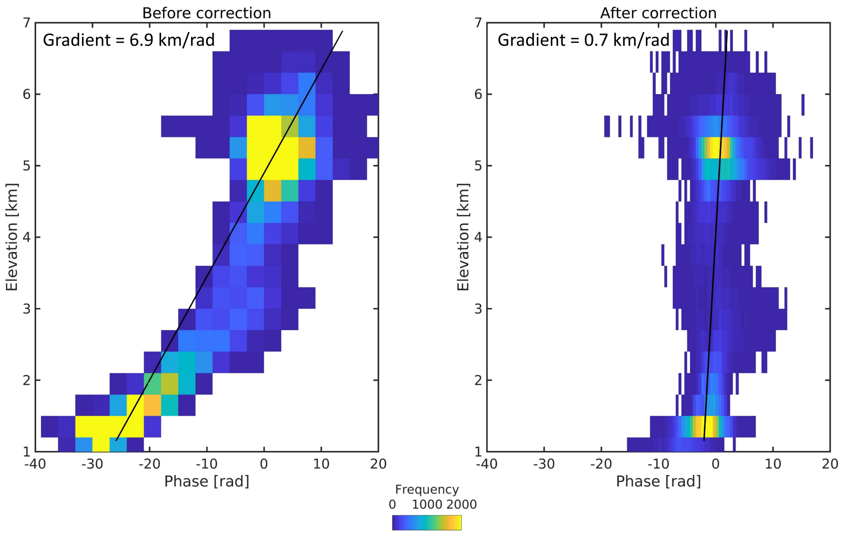

(b) Track 92: 1 Sept 2015

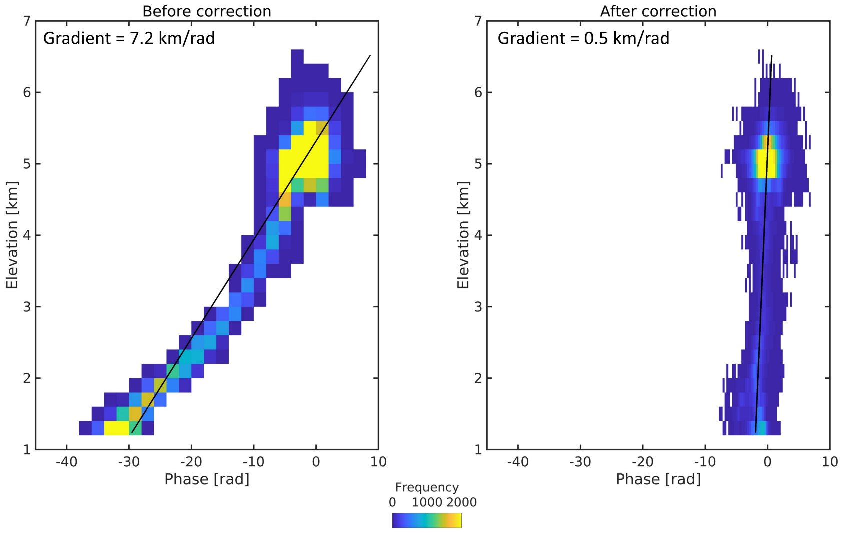

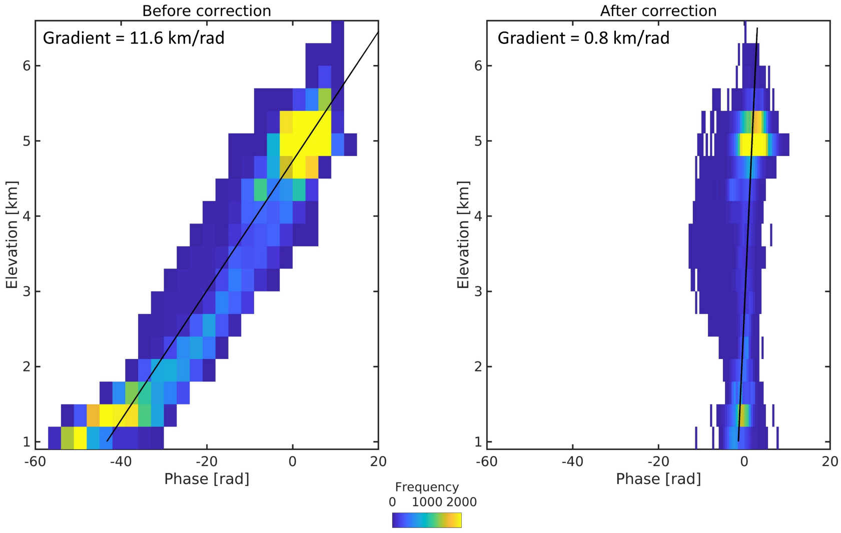

(c) Track 19: 8 Aug 2016

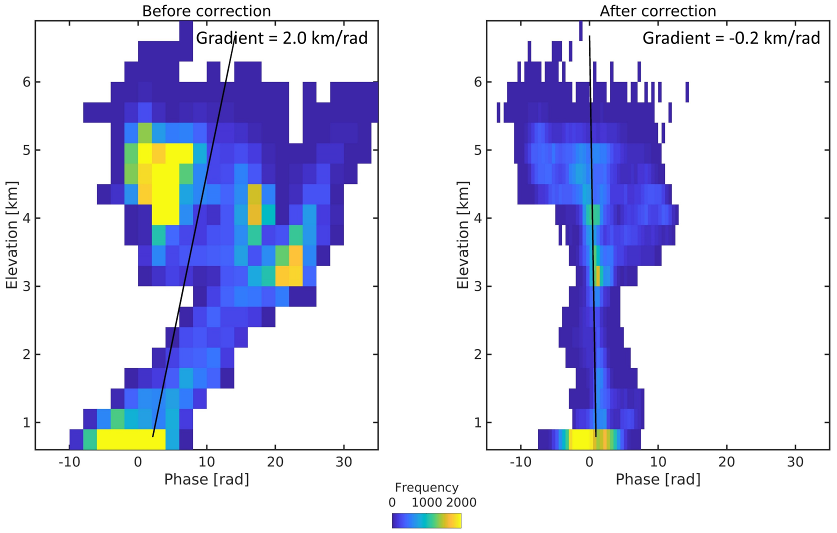

(d) Track 121: 6 Aug 2018

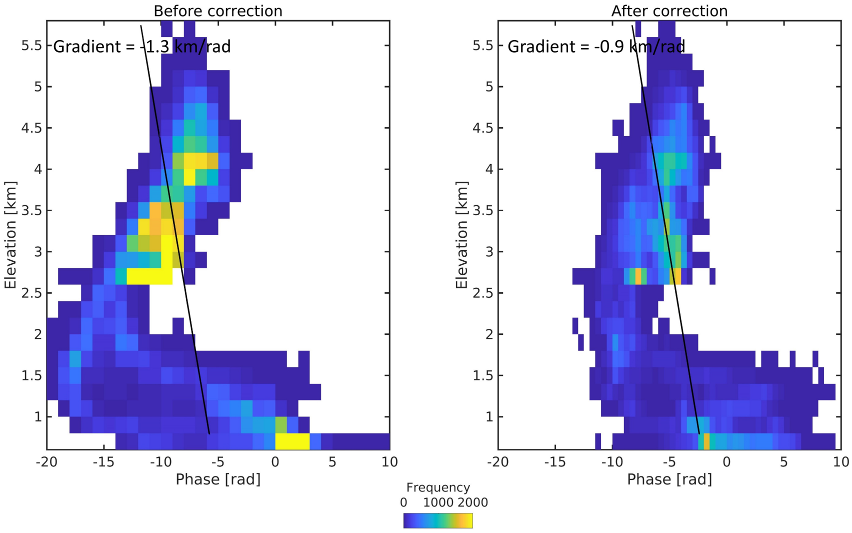

(e) Track 48: 17 Nov 2018

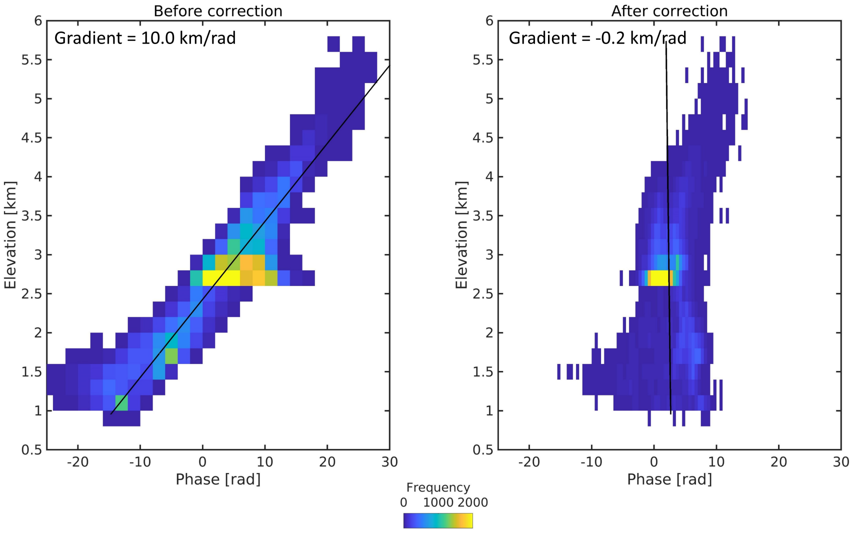

(f) Track 150: 11 Aug 2015

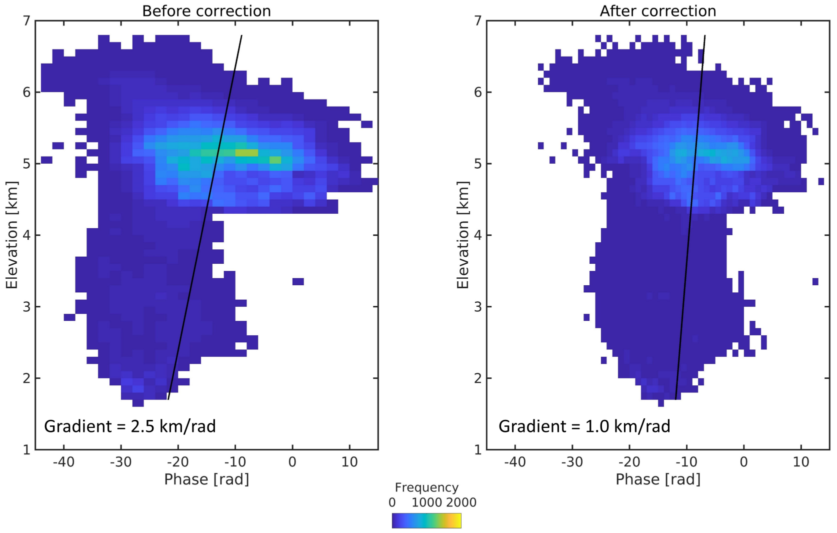

(g) Track 158: 2 Apr 2015

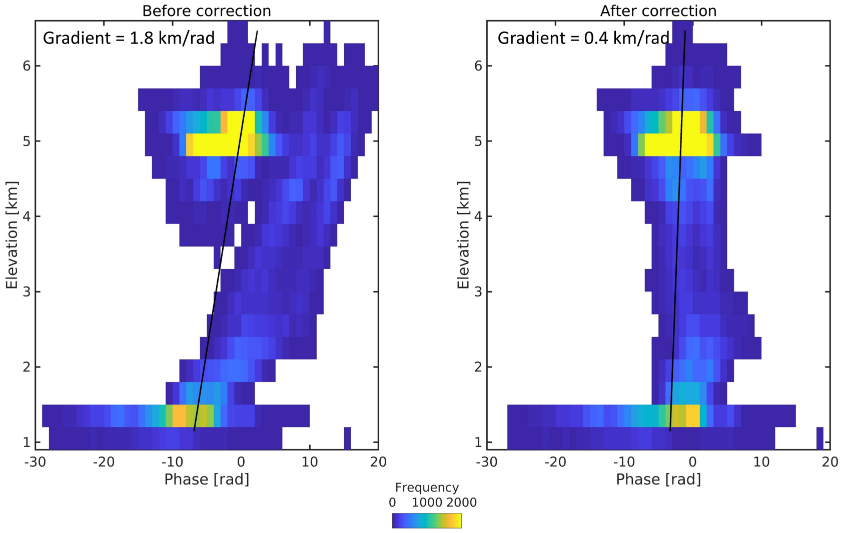

(h) Track 85: 23 Jun 2019

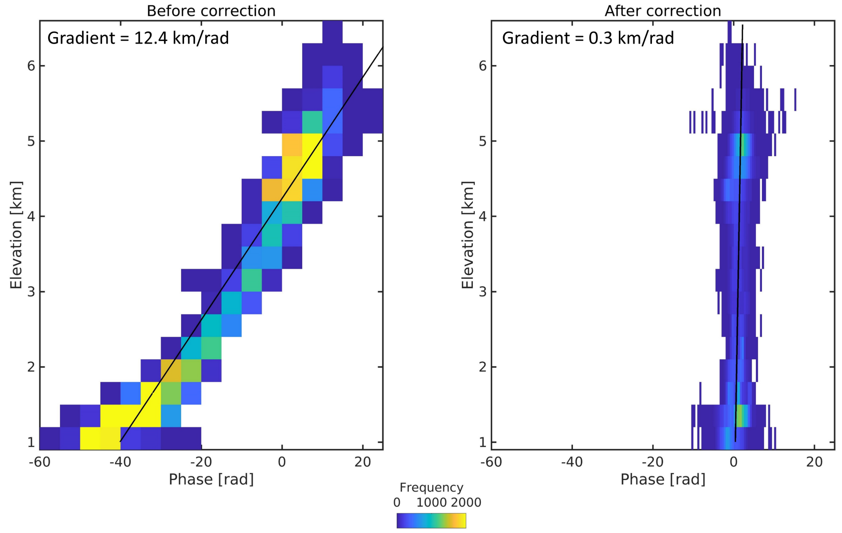

(i) Track 12: 8 Aug 2016

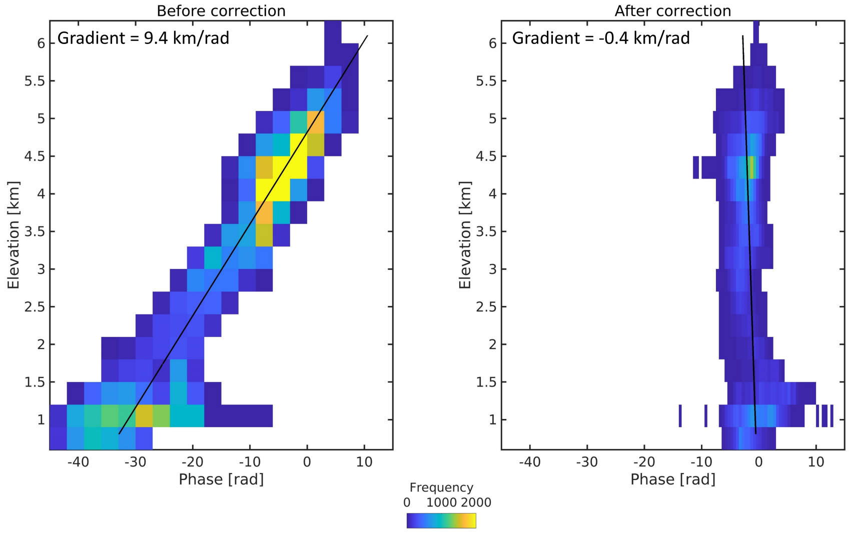

(j) Track 114: 30 Jun 2018

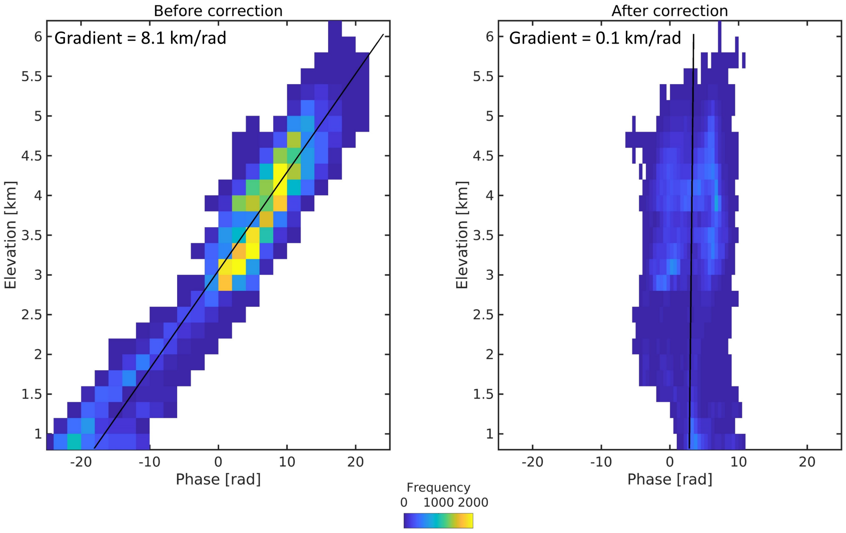

(k) Track 41: 31 Jul 2018

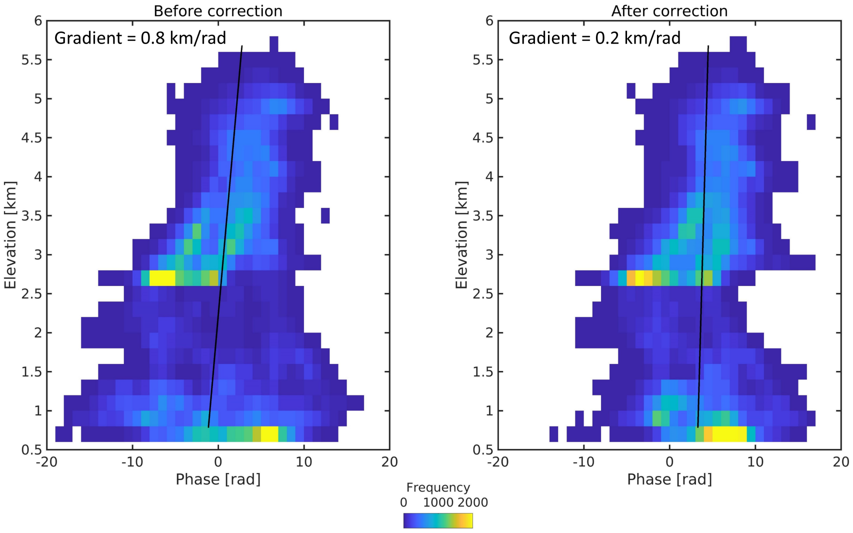

(l) Track 143: 21 Jul 2019

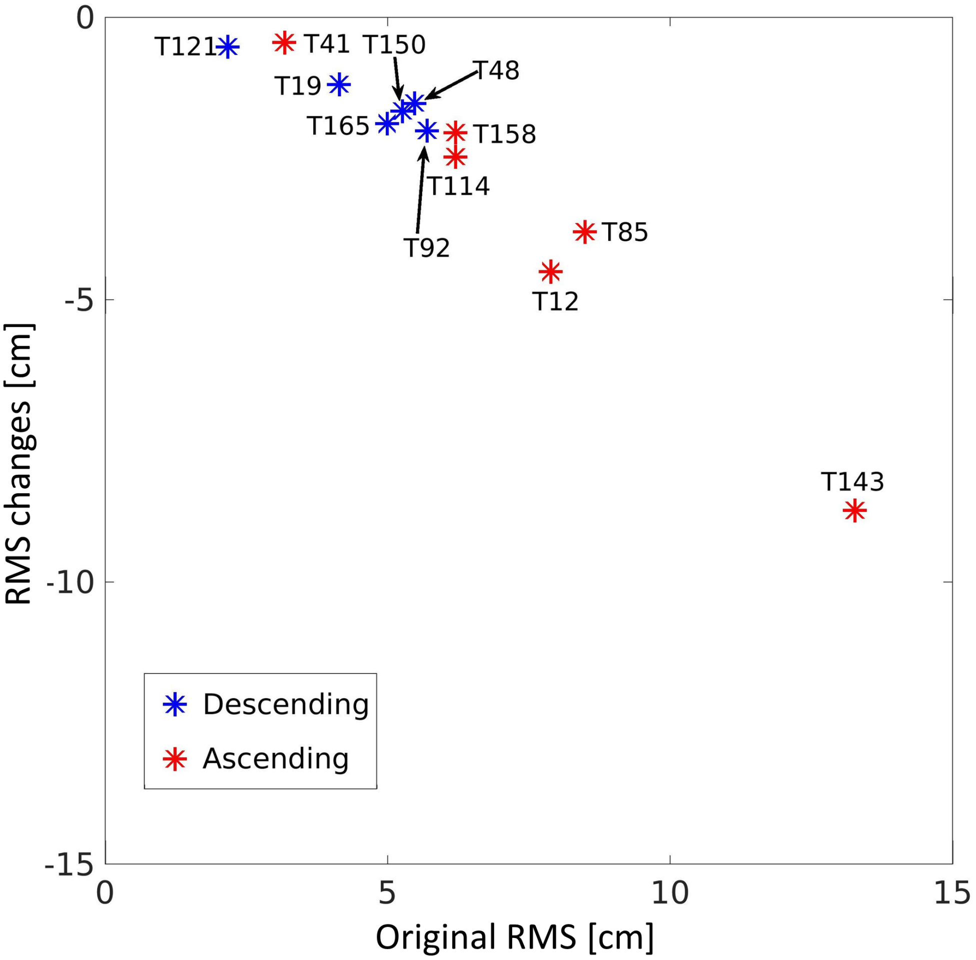

RMS changes [cm]
Original RMS [cm]
0
-5
-10
-15
0
5
10
15
T121
T41
T150
T19
T48
T165
T158
T114
T92
T85
T12
T143
Descending
Ascending

(m)

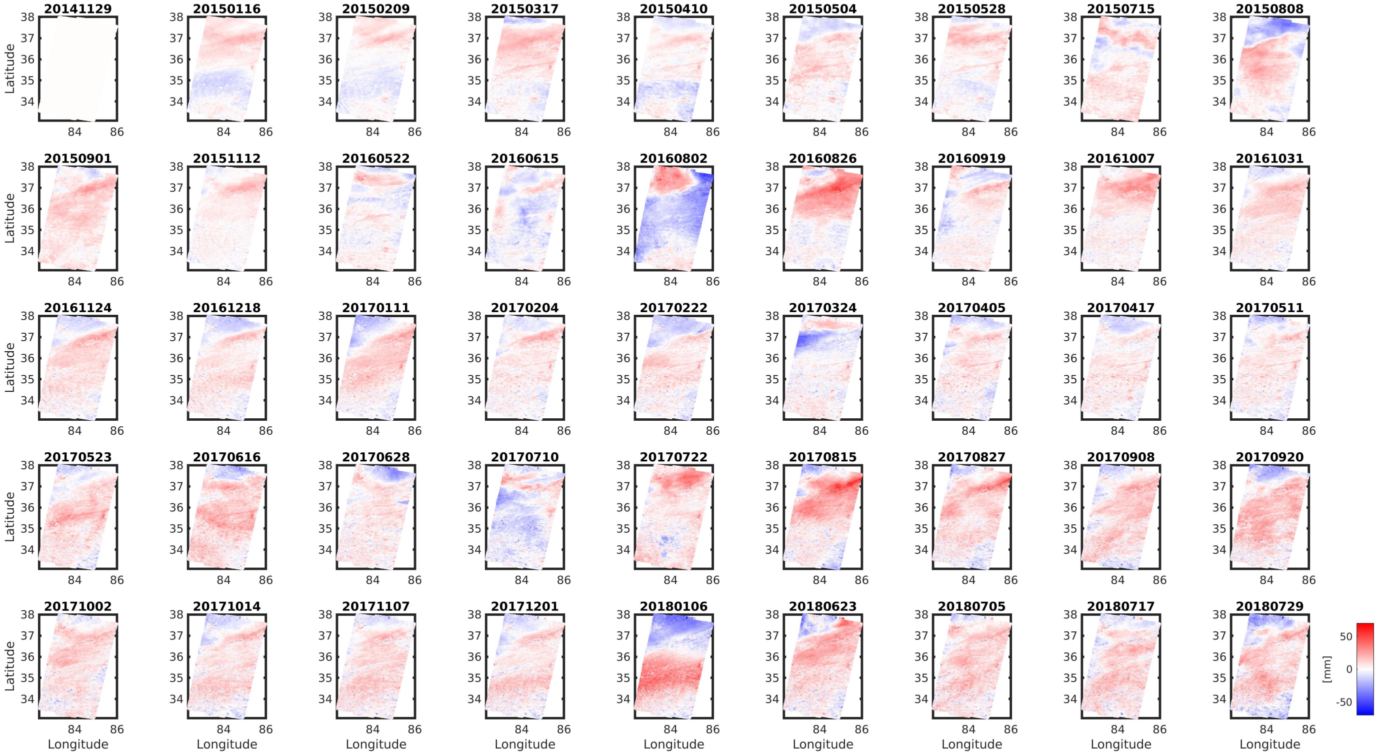

Supplementary Fig. 14: Derived accumulative displacement of descending track 92, showing both short-term and long-term accumulated deformation signals. Positive values indicate the motion towards the satellite, whereas negative values show the motion away from the satellite.

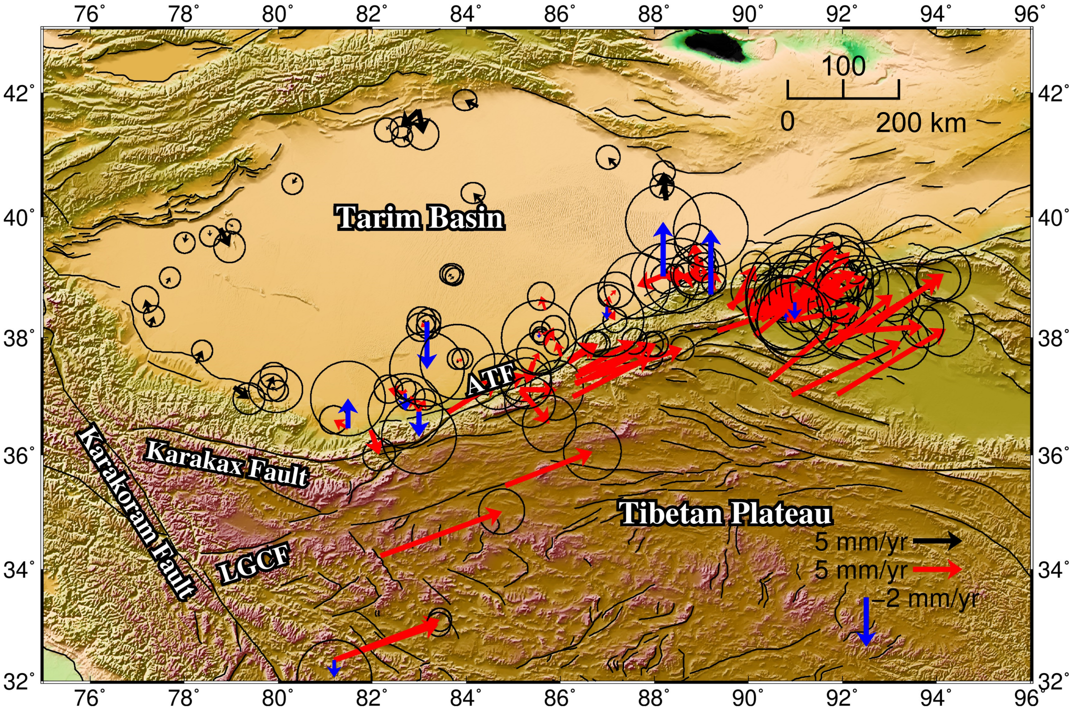

Supplementary Fig. 15: GNSS velocity map. Black vectors show horizontal velocities of the 28 GNSS sites used to define the Tarim Basin reference frame. Red vectors and blue vectors represent horizontal velocities of the 84 available GNSS sites and vertical velocities of the 23 available GNSS observations used to determine the linear planes for each track, respectively. All velocities are plotted in the defined Tarim Basin reference frame at 68% confidence level. ATF = Altyn Tagh Fault. LGCF = Longmu-Gozha Co Fault. The map background shows the elevation of the study region derived from the Shuttle Radar Topography Mission (SRTM) 3-arc seconds data.

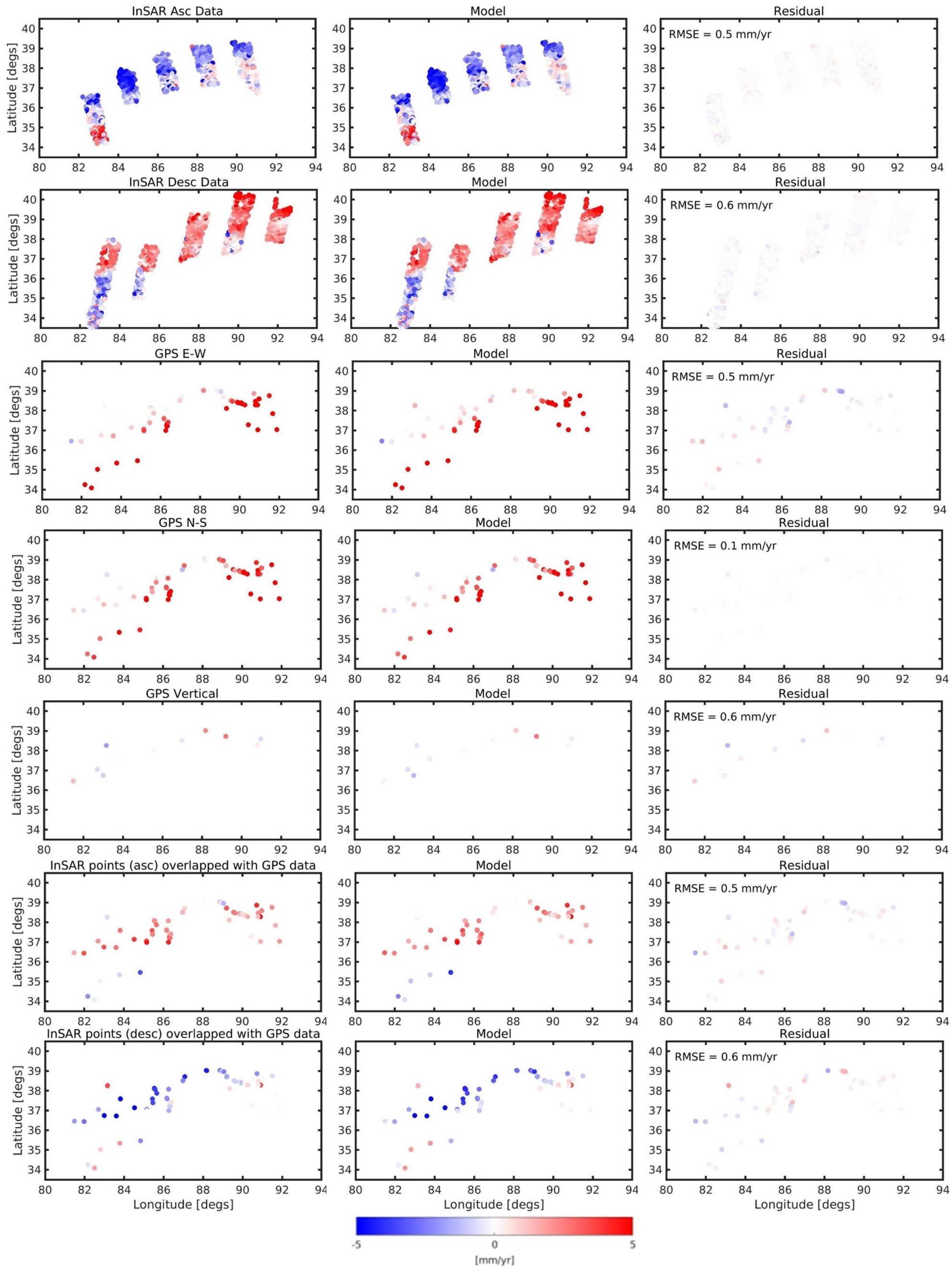

Supplementary Fig. 16: The fit of the model to the data points in the inversion for long wavelength trends. Panels in the first and the second rows show the fit of the model to the InSAR points selected from the overlapping region between adjacent tracks; Panels in the third to the fifth rows indicate the fit of the model to the GNSS data; Panels in the last two rows show the fit of the model to the InSAR points covered by the GNSS sites in ascending and descending, respectively.

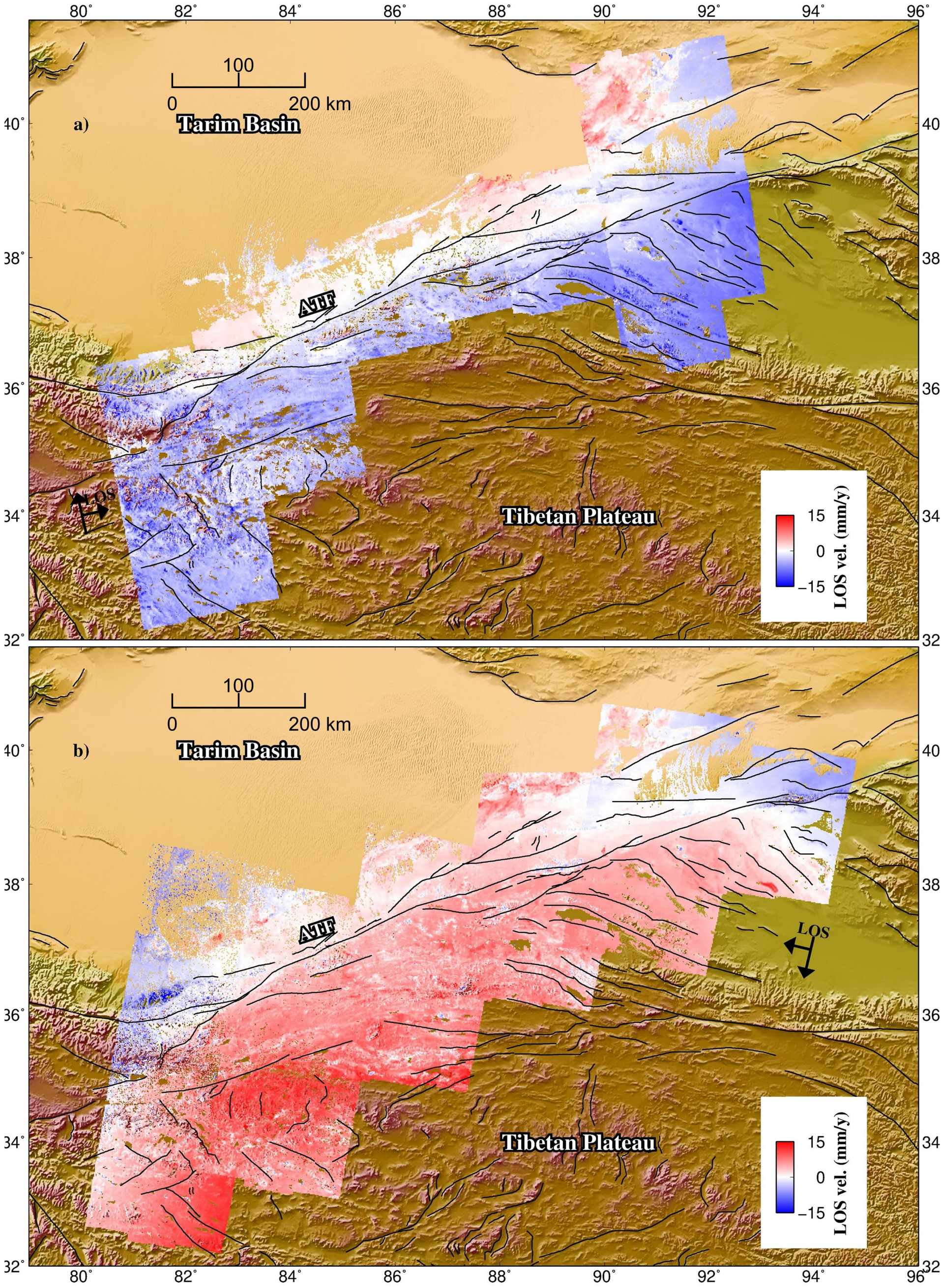

Supplementary Fig. 17: Mosaicked InSAR line-of-sight (LOS) velocity fields of ascending (a) and descending (b) in the Tarim reference after removing the inverted linear planes from each track. Positive values indicate motion towards the satellite. ATF = Altyn Tagh Fault. The map background shows the elevation of the study region derived from the Shuttle Radar Topography Mission (SRTM) 3-arc seconds data.

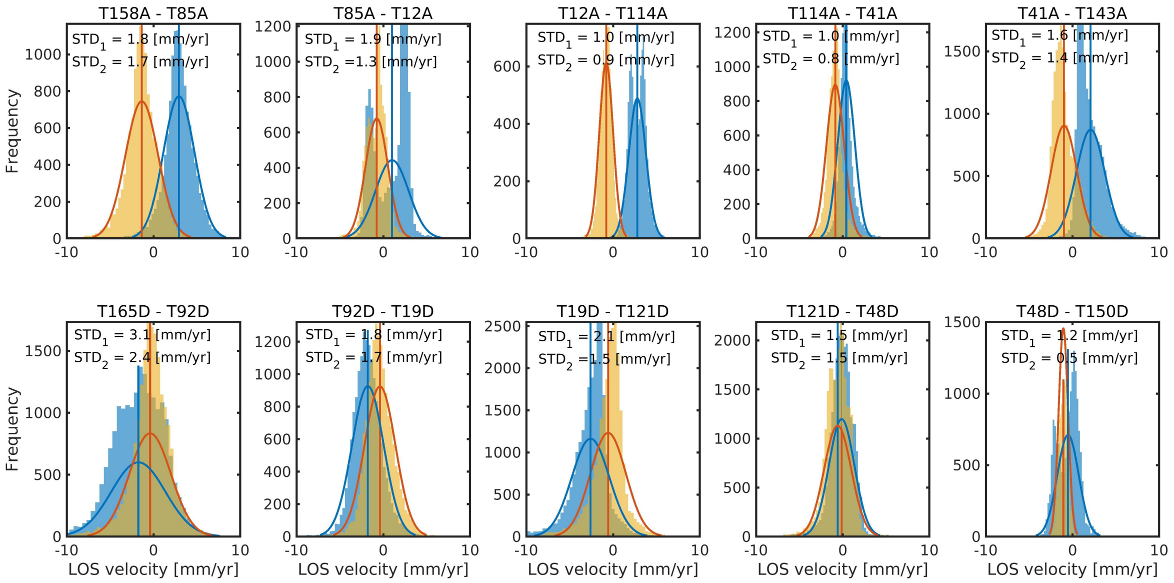

Supplementary Fig. 18: Comparison of the line-of-sight (LOS) velocity differences in the overlapping region between adjacent tracks before and after velocity mosaicking. Blue histograms show the original differences along with the Gaussian distribution fit (blue curves), whereas the yellow-orange colour features the differences after removing inverted linear planes. The blue and orange lines indicate the mean value of the corresponding Gaussian distribution. STD_1 and STD_2 show the standard deviation of each estimated Gaussian distribution before and after velocity mosaicking, respectively. The mean of each is expected to be non-zero due to the different incidence angles.
